# Identification of plant resistance inducers and evaluation of genotype receptivity for carrot protection against Alternaria leaf blight

**DOI:** 10.3389/fpls.2025.1513301

**Published:** 2025-03-05

**Authors:** Valérie Le Clerc, Sitti Anlati Moussa, Anita Suel, Claude Emmanuel Koutouan, Angelina El Ghaziri, Matthieu Gaucher, Marie-Noelle Brisset, Mathilde Briard, Emmanuel Geoffriau

**Affiliations:** ^1^ Institut Agro, Université d’Angers, INRAE, IRHS, SFR 4207 QUASAV, Angers, France; ^2^ INRAE, Institut Agro, Université d’Angers, IRHSR 4207, SF, QUASAV, Angers, France

**Keywords:** *Daucus carota* L., quantitative real-time PCR (qPCR), defence gene, plant resistance, *Alternaria dauci*, crop sustainability

## Abstract

The use of biopesticides represents an alternative strategy to synthetic chemical products for crop protection. To promote their adoption and effective use by growers, it is crucial to understand their modes of action and the optimal conditions for their application in crops, including their compatibility with specific varieties. Through a series of greenhouse experiments, this study describes the development and validation of a robust molecular diagnostic tool for enabling the evaluation of defence gene activation. The results identified plant resistance inducers (PRIs) among biopesticide products capable of protecting carrots against Alternaria leaf blight. By applying a PRI to a range of carrot varieties exhibiting varying levels of resistance to *Alternaria dauci*, preliminary findings on plant receptivity suggest that the efficacy of PRIs in conferring protection is highly dependent on the treated variety. Two distinct genotype-dependent effects were observed: sensitivity to the PRI and an enhancement of resistance. This study offers new insights into optimising biopesticide use in carrot cultivation.

## Introduction

Although the use of biopesticides is widely recognised as a promising alternative for reducing reliance on synthetic plant protection products, their implementation remains challenging across different pathosystems. To enable large-scale adoption in agricultural production, it is essential to provide growers and extension services with practical guidelines for the effective use of these alternative solutions. Developing reliable recommendations requires a thorough understanding of the products’ mode of action and the influence of the environment and agricultural practices on their efficiency.

Among the various biopesticide products, plant resistance inducers (PRIs) enhance a plant’s ability to face disease attacks by activating its innate defence mechanisms (Burketova et al., 2015). PRIs are diverse in nature and origin, encompassing animal, plant, microbial, or mineral sources. When applied to plants, they are intended to trigger the transcription of specific defence-related genes and promote the production of proteins, hormones, or specialised metabolites, allowing the plant to defend against a future attack of the targeted pathogen. However, their efficacy can be influenced by several factors (Walters et al., 2013) with plant genotype being a critical determinant. For instance, in tomato, the application of *Trichoderma atroviride* and *Trichoderma harzianum* has been shown to enhance systemic resistance against *Botrytis cinerea* in a plant genotype-dependent manner (Tucci et al., 2011).

*Alternaria dauci* (Ad) is the primary pathogen responsible for the most severe foliar disease affecting carrot (*Daucus carota* L.) crops. To explore alternative protective strategies against this disease, known as Alternaria leaf blight (ALB), we conducted a preliminary assessment. In this study, we assessed the protective efficacy of 10 biological plant protection products (bioPPPs) and one fungicide. Our results indicated that seven of these products demonstrated a high level of effectiveness in protecting the plant against the disease (Moussa et al., 2024). Three of these bioPPPs are formulated with microorganisms, specifically two species of *Bacillus* (the active ingredient in Rhapsody^®^ and Sonata^®^) and one *Trichoderma*. *Bacillus* and *Trichoderma* are among the most extensively studied biopesticide agents in agriculture, renowned for their ability to control a wide range of pathogens and exhibit diverse activities against fungal species (Scortichini, 2022; Tyśkiewicz et al., 2022). These mechanisms include i) direct effects on fungi, such as antibiosis, hyperparasitism, or competition for nutrients or space; ii) interference with pathogenesis processes, including surfactant activity on the plant epidermis to inhibit fungal spore adhesion and germination or production of enzymes that obstruct fungal hydrolytic functions; and iii) indirect effects by eliciting plant resistance (PRI effect). Vacciplant^®^ is a bioPPP based on laminarin, a beta-glucan extracted from the brown alga *Laminaria digitata*, which is well-known for providing protection to wheat against *Zymoseptoria tritici*. This protection is mediated by the elicitation of plant defence genes, complemented by a direct antifungal effect likely attributable to additives (De Borba et al., 2022). Additionally, Vacciplant^®^ has demonstrated efficacy in controlling downy mildew (Taibi et al., 2022). LBG 01F34^®^ is registered as a fungicide for vines and exerts both direct effects on mildew and indirect effects via its PRI activity. Its active substance, phosphite, is capable of stimulating or priming the plant immune response in various species, including potato against *Phytophthora infestans* (Machinandiarena et al., 2012) and apple against three major bioagressors: *Venturia inaequalis*, *Erwinia amylovora*, and *Dysaphis plantaginea* (Gaucher et al., 2022). Helioterpen^®^ soufre is a liquid sulphur co-formulated with pine terpenes registered as a biofungicide with a broad spectrum of activity. Finally, Bion^®^ is a fungicide that, while not registered as a bioPPP, is certainly the most well-known commercial PRI. Formulated with acibenzolar-*S*-methyl (ASM), it provides protection to plants against a variety of bacterial and fungal pathogens (Cole, 1999; Małolepsza, 2006; Santos et al., 2022). Notably, Bion^®^ conferred the highest level of protection to the susceptible carrot genotype.

To summarise, these seven products, when used under controlled conditions, yield promising results. Even if most of them are supposed to be PRI, their modes of action are not or only partially characterised and exclusively on other plant species. However, as highlighted earlier, understanding how the products work in the target plant—here, carrot—and identifying the factors that may influence their efficacy are critical for their effective application. Numerous plant defence genes coding for instance for pathogenesis-related (PR) proteins, cell wall modifications, antioxidant mechanisms, specialised metabolic pathways, and signalling pathways have been described in the literature. Conducting experiments under varying conditions to investigate factors influencing product efficacy without knowing which genes to target in the analyses can quickly become exceedingly tedious and time-consuming. To address this challenge, Dugé De Bernonville et al. (2014) developed an innovative system enabling the simultaneous study of multiple defence pathways of the apple tree (patent: WO/2011/161388). This molecular diagnostic tool allows for the evaluation of the expression levels of 28 target genes involved in different defence pathways at a given time. In this study, we propose to adapt this tool to carrot as part of an extension of this patent.

The objectives of this study are therefore i) to describe the development of a biomolecular tool for assessing the expression of defence genes in carrot, ii) to identify the “true” carrot PRIs among the seven efficient products, and iii) to evaluate potential genotype-dependent differences in response.

## Materials and methods

### Global framework of the study

The original apple chip device using the qRT-PCR technology consists of a ready-to-use 96-well microtitration plate containing the primer pairs targeting 31 apple genes including 28 defence genes and three reference genes in triplicate. The adaptation of the apple tool to the carrot species within the framework of the extension of the WO/2011/161388 patent required maintaining the same design and overall functionality of the chip. To analyse all the genes simultaneously, it is particularly important that all genes can be amplified under the same experimental conditions. Their expression must be stable when the experimental conditions are identical. Their expression must be informative, which means that it should show differences when the experimental conditions change, for example, from one treatment to another. Finally, they must cover all the metabolic pathways described in the apple patent.

To select the most appropriate genes, we examined the carrot genome for defence genes that are homologous to those found in the apple tree, as well as analysed a set of genes that have previously been identified as being involved in carrot resistance to Ad (Koutouan et al., 2018).

**Figure 1:**
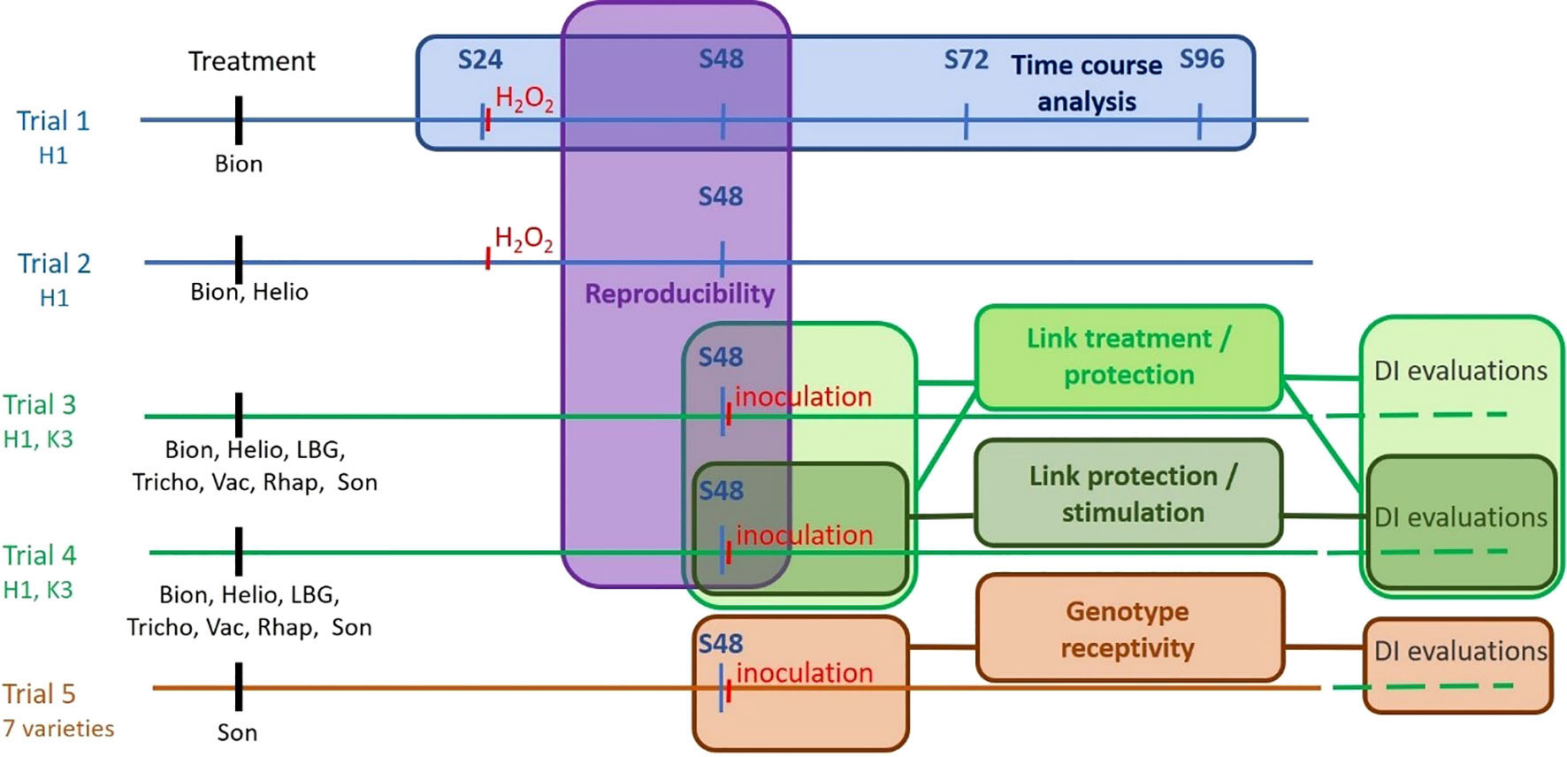
Global diagram of the five trials supporting the study. Trials performed in year 1 are in blue, in year 2 in green, and in year 3 in brown. Genotypes used in the different trials are mentioned under the trial label. S24, 48, 72, and 96 are sampling dates 24, 48, 72, and 96 hours post-treatment (hpt), respectively. DI, disease index. The coloured boxes indicate the objective of each trial. Bion, Bion^®^; Helio, Helioterpen^®^ soufre; LBG, LBG 01F34^®^; Tricho, Trichoderma strain; Vac, Vacciplant^®^; Rhap, Rhapsody^®^; Son, Sonata^®^.

To determine the best experimental conditions, to identify PRI on carrot, and to evaluate potential genotype-dependent differences in response, five independent greenhouse trials (**Figure 1**) were carried out over 3 years in Angers, France. The first two trials focused on the development of the biomolecular tool, while the last three were dedicated to its validation and exploration of biological questions regarding the effects of bioPPP or plant genotypes.

### Plant material

The plant genotypes were selected according to their resistance to Ad: H1 is a highly susceptible French S3 line from the HM Clause (La Bohalle, France) breeding program. K3 and I2 are two highly partially resistant Asian-type S2 lines developed by the Institute of Research in Horticulture and Seeds (IRHS) (Angers, France) (Le Clerc et al., 2015). Seven commercial carrot varieties currently used by the French growers and recommended by the extension service Invenio (Ychoux, France) were also selected based on their resistance levels to Ad to encompass a range of susceptibility: susceptible (S), intermediate (I), and resistant (R) genotype. These varieties include Presto (S), Texto (I), Soprano (I), Bolero (I), and Maestro (I), which are five hybrids from Vilmorin (La Ménitré, France), as well as Romance (R) and Brillyance (R), two hybrids from Nunhems (Beaucouzé, France).

### Plant management

Five-litre pots were filled with Traysubstrat from Klasmann-Deilmann^®^ (Geeste, Germany). For each genotype, 10 seeds were sown per pot. After 30 days, only seven seedlings were retained in each pot. Each pot containing seven plants was considered as one replicate per treatment modality (variety × treatment). For the first two trials (1 and 2), three replicates were conducted, while for the last three trials (3, 4, and 5), four replicates were performed. The photoperiod was set to 16 hours of light and 8 hours of darkness, with temperature maintained at 20°C ± 2°C during the day and 18°C ± 2°C at night.

### Application of biopesticides

Two days before bioPPP treatment, pots were grouped by modality (bioPPPs or controls) under mini tunnels to prevent cross-treatment contamination. At the 4–5 leaf stage, plants were sprayed once with bioPPPs, Bion^®^, or water (15 mL per pot) until runoff, using a spray gun (Deltalyo Aeryo-1.4 model) connected to a compressor. The list of products and their applied doses is provided in **Table 1**.

**Table 1:** Products and controls used in trials, active ingredient, dosage, and suppliers.

Treatment	Active ingredient	Maximum certified dose	Company–supplier
Bion^®^ 50 WG	Acibenzolar-*S*-methyl	0.08 kg/ha	Syngenta France AS
Helioterpen^®^ soufre	Sulphur + co-formulation based on terpene derivatives from pine	6 L/ha	Action Pin
LBG 01F34^®^	Potassium phosphonate	2 L/ha	De Sangosse
Trichoderma	Trichoderma strain	10^4^ conidia/mL	FungiSem team research (IRHS)
Vacciplant^®^	Laminarin	2 L/ha	Goëmar
Rhapsody^®^	*Bacillus subtilis* str. QST 713	10 L/ha	Bayer
Sonata^®^	*Bacillus pumilus* QST 2808	10 L/ha	Bayer
Water control			
H_2_O_2_	Hydrogen peroxide	5 mg/L^a^	

a 30% solution.

### Hydrogen peroxide treatment or inoculation with *A. dauci*

In Trials 1 and 2, in order to rigorously reproduce the experimental conditions described by Dugé de Bernonville et al. (2014), carrot leaves were sprayed with hydrogen peroxide (40 mM) at 24 hours post-treatment (hpt). According to these authors, this treatment was designed to simulate the stress caused by a pathogen attack. In Trials 3, 4, and 5, we opted to apply the pathogen itself rather than relying on a simulation. For this, the inoculum of the Ad P2 strain (moderate aggressiveness) was prepared following the method described by Pawelec et al. (2006). Briefly, a fungal suspension in water with Tween 20 (0.05%) was filtered through two layers of cheesecloth, and the conidial concentration was adjusted to 4–5 × 10^3^ conidia/mL. Inoculation was performed 48 hpt using a hand-held sprayer. Tunnels were closed for 2 days to promote fungal infection. After pathogen inoculation, the greenhouse temperature was maintained at 23°C during both night and day, with humidity controlled using a fogging system.

### Disease evaluation and statistics

Trials 3–5 were carried out over two different years and different season times. The speed of ALB symptom evolution varies from one trial to another, particularly depending on the ambient temperature. To trigger an evaluation operation for a given trial, indicator plants were used as performed by Le Clerc et al. (2015). These were plants of the Presto cultivar, a very susceptible commercial variety. They were observed three times a week. When the disease started to develop on these indicator plants, the first evaluation was triggered. When disease symptoms increased by at least 1 point compared to the previous evaluation, a new evaluation was undertaken.

In Trials 3 and 4, the seven products, along with water as a control (factor product), were applied on each of the two genotypes (H1 and K3) four times (factor repetition). The disease index (DI) was evaluated twice, 26 and 39 or 18 and 35 days after inoculation (factor time). Each pot was assigned a score based on a visual scale of symptoms caused by Ad on carrot leaves, where 0 represented symptomless plants and 9 corresponded to completely blighted plants as previously described by Le Clerc et al. (2015) (response variable score). To analyse the two sets of data (scoring dates 1 and 2) within a single analysis and thereby increase statistical power, it was important to avoid bias related to the non-independence of variables based on these so-called “repeated” measures. To achieve this, as required in the lmer function of the *lmerTest* package of R, an identifier (factor id) was created to track a pot throughout the experiment. Thus, a score assigned to a given pot during the first evaluation was linked to the score of that same pot during the second evaluation. A study for each genotype was realised using a linear mixed model with factors time and product as fixed, and id and repetition as random. After the postulates were checked (the residual normality and homoscedasticity, and the variance homogeneity of the dataset), the lmer function of the *lmerTest* package of R was used to apply this model (**Supplementary Material 1**). When the interaction was not significant, an additive model was used. Pairwise comparisons to assess the significance of differences between treatments were conducted for each genotype using Tukey’s test (performed with the *emmeans* package in R). As significant differences were observed only in comparison with water, a Dunnett’s test was applied to compare all products against water (the control modality). The results of Dunnett’s test are presented using radar chart plots.

In Trial 5, the DI was evaluated six times (at six scoring dates from 14 to 53 days after inoculation) across four replicates (tunnels). The area under the disease progression curve (AUDPC) was calculated from the disease intensities at all scoring dates using the *AUDPC* package in R. This provides a synthetic value representative of the disease severity over the entire epidemic for each variety and the two treatments: water and Sonata^®^ (**Table 1**) for each replicate. Analyses of variance (ANOVAs), along with pairwise comparisons using Tukey’s test between treatments, were performed for each variety (**Supplementary Material 2**).

### Development of a biomolecular tool adapted for carrot

In addition to the genes present on the original apple chip, we decided to evaluate 13 candidate genes involved in carrot defence mechanisms, based on previous results (Koutouan et al., 2018; Lecomte et al., 2012). Most of these genes are implicated in the terpenoid biosynthesis pathway. We selected a total of 44 genes, including the three reference genes, 28 genes homologous to those present on the apple biomolecular tool, and 13 genes potentially involved in carrot resistance to *Ad*.

**Figure 2:**
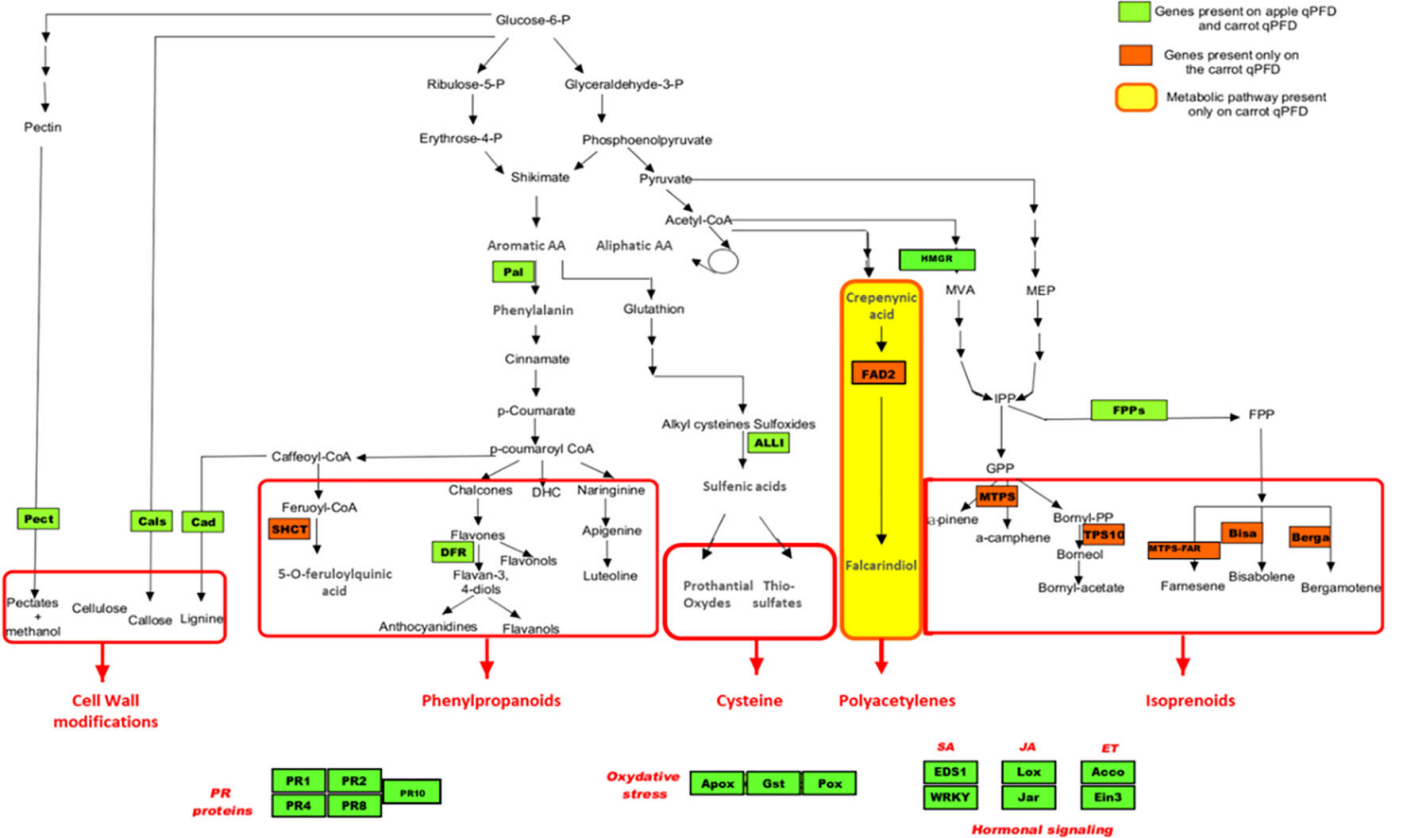
Representation of the 29 genes (green and orange boxes) of the biomolecular tool on defence pathways. PR, pathogenesis related; SA, salicylic acid; JA, jasmonic acid; ET, ethylene.

**Figure 3:**
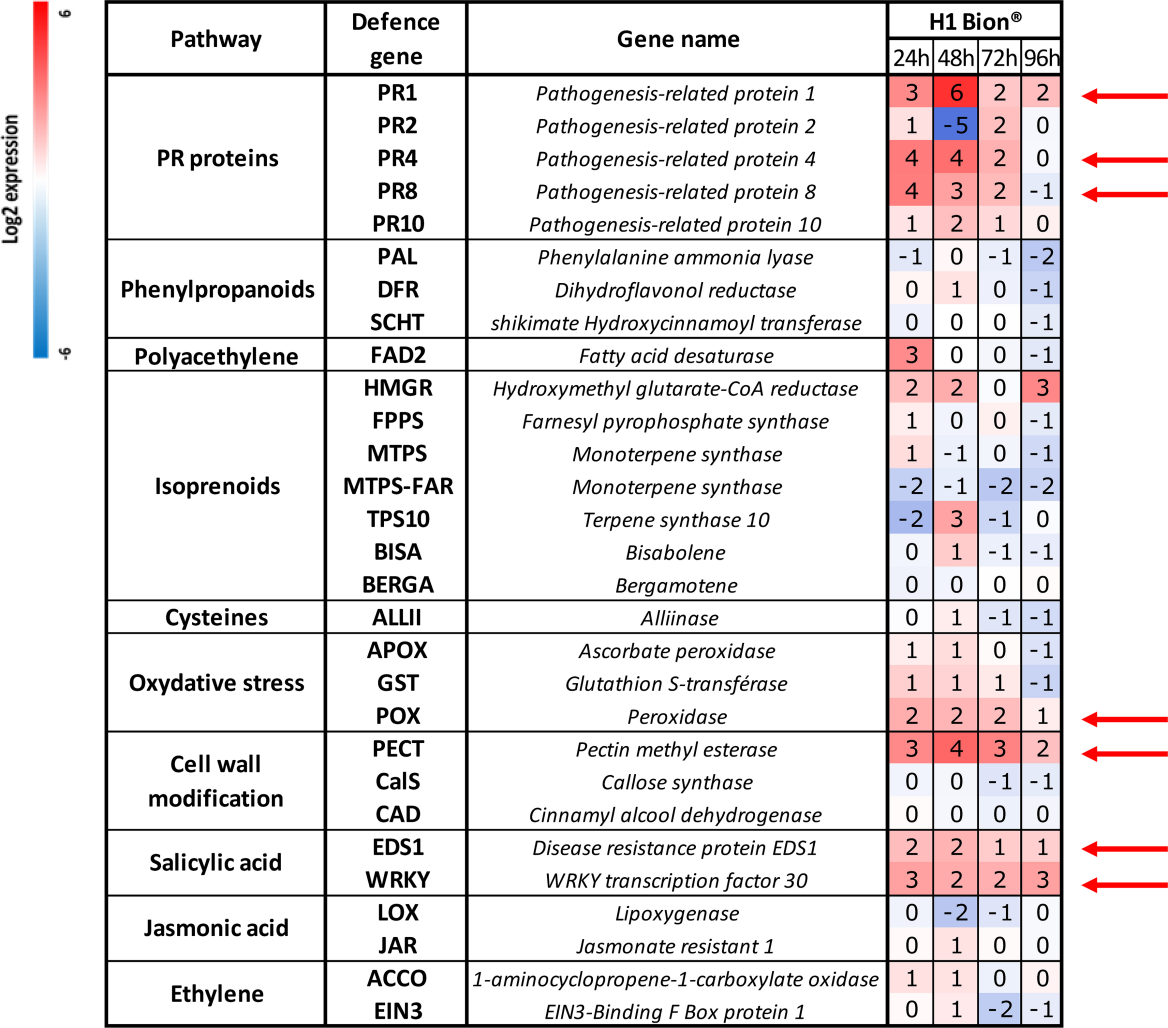
Relative defence gene expression of the genotype H1 at different times (24, 48, 72, and 96 hours) after spraying with Bion^®^ in Trial 1. At 24 hpt, the negative controls were samples treated with water, while for other sampling times, negative controls were samples treated with hydrogen peroxide. Each value is the average result of the three technical replicates of the pool of six leaves (i.e., 2 leaves of 1 plant per replicate × 3 biological replicates). Red arrows point out the seven genes with a continuous and highest upregulation between 24 and 72 hpt.

For the genes homologous to those in apple, the cDNA sequences described in patent WO/2011/161388 were used as reference sequences. Thirty-one apple sequences were loaded into the Geneious software (v.10.2.3; https://www.geneious.com), and each sequence was used as a query in a *tblastx* homology search against the *D. carota* genome (GeneBank number LNRQ00000000.1). The best *D. carota* hits were then selected. For the 13 genes not present on the apple chip, sequences were selected arbitrarily, one after the other, from the National Center for Biotechnology Information (NCBI) database. Forward and reverse primers (Eurofins, Paris, France) for all candidate sequences were designed using the “Design new Primers” tool in Geneious (v.19.2.3) with the following parameters: primer lengths of 18 to 22 pb, product lengths of 100 to 200 pb, Tm values ranging from 62°C to 65°C, and GC contents between 50% and 60%. The final list of defence genes and defence pathways covered by the carrot chip is provided in **Figures 2** and **3**.

To ensure that the tool works effectively in the future, regardless of the variety, environment, or plant age—i.e., under any experimental conditions—the specificity and efficiency of the primers were studied using a panel of cDNA templates as diverse as possible: three genotypes (H1, K3, and I2), two environments (tunnel and greenhouse), Ad-inoculated plants or not, plants treated with water or various bioPPPs, and various sampling times (6, 24, 36, 48, 72, and 96 hours after treatment or 8 days after treatment). Leaf samples were immediately freeze-dried in liquid nitrogen.

Total RNA was extracted from each sample using the MACHEREY-NAGEL NucleoSpin^®^ RNA PLANT kit (Macherey-Nagel, Düren, Germany) with slight modifications concerning the centrifugation times, all of which were doubled. RNA concentration was determined using a NanoDrop™ One Spectrophotometer (Thermo Fischer Scientific, Saint-Herblain, France). First-strand cDNA was synthesised with 1 µg of total RNA using M-MLV reverse transcriptase (Promega, Madison, WI, USA) and oligo(dT)_15_ (Promega) in a 40-µL reaction volume. DNA contamination was assessed by PCR using EF1α primers that flank an intron to discriminate the size of amplicons, and the results were visualised by electrophoresis on a 1.5% agarose gel.

Quantitative real-time PCR was performed on a CFX384 Touch Real-Time PCR System (Bio-Rad, Hercules, CA, USA) using GoTaq^®^ qPCR Master Mix (Promega). A 10-µL reaction mixture consisted of 5 µL GoTaq^®^, 2 µL pooled cDNA, 2 µL mixed primers (at the desired concentration), and 1 µL RNase-free water. The amplification conditions were as follows: 95°C for 5 min, followed by 39 cycles of 95°C for 30 seconds and 60°C for 60 seconds. The melting curve was analysed to assess primer specificity, and amplification efficiency was calculated across a cDNA dilution range (1/4) using the Auto Efficiency function from CFX Manager Software (Bio-Rad).

### Validation and application of the biomolecular tool for the pathosystem carrot/*A. dauci*

Regardless of the modality, qRT-PCR analyses were performed on leaves sampled as described in **Figure 1**: 24 hpt (S24), 48 hpt (S48), 72 hpt (S72), and 96 hpt (S96) in Trial 1. In subsequent trials, based on the results of Trial 1, samplings were conducted at 48 hpt (S48). In Trial 1, hydrogen peroxide treatment was applied immediately after S24. In Trials 3–5, inoculation with Ad P2 strain was performed immediately after S48. Regardless of the sampling date, the two intermediate leaves from one plant per replicate of each genotype were pooled, immediately frozen in liquid nitrogen, and stored at −80°C until further use. Subsequently, leaf samples were ground in liquid nitrogen using a mortar and pestle. RNA extraction, reverse transcription, and qRT-PCR were performed as previously described, with primers used at the optimal concentrations determined during the tool development phase.

In Trial 1, the four sampling dates (S24 to S96) were selected to identify the optimal time for analysing gene expression after treatment. At each sampling point, relative changes in defence gene expression (log2 ratio) were calculated using the 2^−ΔΔCt^ method, with normalisation performed using three reference genes (Schmittgen and Livak, 2008). The calibrator was water control for the S24 samples and the hydrogen peroxide treatment for the subsequent sampling points (S48, S72, and S96).

To evaluate the reproducibility of gene expression across trials, the S48 results from Trials 1, 2, 3, and 4 were compared. Hydrogen peroxide treatment was used as the calibrator in Trials 1 and 2, while water served as the calibrator in Trials 3 and 4.

## Results

### Development and validation of the biomolecular tool for carrot

#### Primers and amplification efficiency

A total of 36 primer sets were validated, as the entire melting curve analysis for each showed a single peak. For these genes, qRT-PCR efficiency ranged from 89.5% to 109.7% with correlation coefficient (R^2^) varying from 0.899 to 0.999. The biomolecular tool employs a 96-well qPCR format, enabling the simultaneous monitoring of up to 32 genes in triplicate. Therefore, the 29 most informative genes, selected based on their induction profiles in carrot and similar melting temperatures (Tm), along with three reference genes, were incorporated into the final chip design (**Figure 2**). Among these 32 genes, seven are specific to the carrot system: one encoding a shikimate *O*-hydroxycinnamoyltransferase enzyme (*SCHT*) involved in the upstream of the phenol pathway, five encoding terpene synthases (*BISA*, *BERGA*, *MTPS*, *MTPS-FAR*, and *TPS10*), and one encoding a fatty acid desaturase (*FAD2*) in the polyacetylene pathway.

#### Expression of defence genes over time following Bion^®^ application

Analysis of relative defence gene expression in the H1 carrot genotype during Trial 1 (**Figure 3**) revealed that Bion^®^ significantly altered the expression levels of numerous defence-related genes in this susceptible genotype.

The most pronounced and sustained upregulations, persisting at least 72 hours, were identified in seven genes: *PR1*, *PR4*, and *PR8* (pathogenesis-related genes); *POX* (involved in oxidative stress); *PECT* (associated with cell wall modification), and *EDS1* and *WRKY* (linked to salicylic acid signalling pathway). The peak expression levels for *PR1* and *PECT* were recorded at 48 hpt. Other genes were repressed, only weakly sensitive to the application of the product, or showed unstable expression between dates, oscillating between overexpression and repression without a clear trend. Only the seven most overexpressed genes were selected to analyse the reproducibility of the tool.

#### Reproducibility of the gene expression

**Figure 4:**
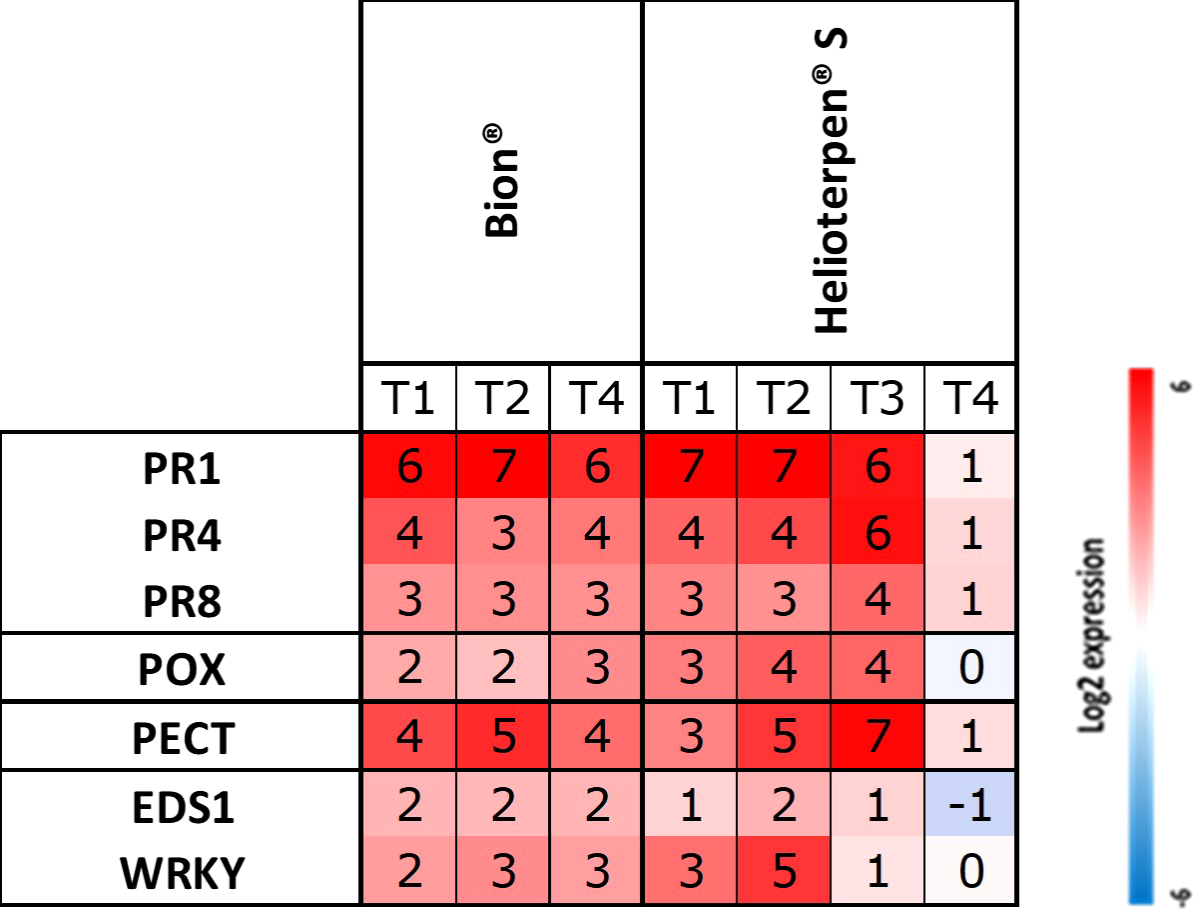
Relative expression of seven defence genes (PR1, PR4, PR8, POX, PECT, EDS1, and WRKY) for the susceptible carrot genotype H1 from three and four independent trials (Trials 1 to 4 called T1, T2, T3, and T4, respectively) 48 hours post-treatment with Bion^®^ or Helioterpen^®^ soufre. Each value represents the means of three technical replicates of six leaf pools (i.e., 2 leaves of 1 plant per replicate × 3 biological replicates) for T1 and T2 and eight leaf pools (4 biological replicates) for T3 and T4. Data were missing for Bion^®^ in Trial 3.

Consistent gene expression profiles were observed across Trials 1, 2, and 4 for the seven genes analysed at 48 hours post-Bion^®^ treatment (**Figure 4**). Similarly, gene expression induction was reproducible across Trials 1, 2, and 3 following Helioterpen^®^ soufre treatment, although variations in intensity were noted, with particularly low expression for the *WRKY* gene in Trial 3. Surprisingly, no gene induction was detected after Helioterpen^®^ soufre treatment in Trial 4.

### Identification of PRIs among products effective for carrot protection against *Ad*

#### Protection efficacy

**Figure 5:**
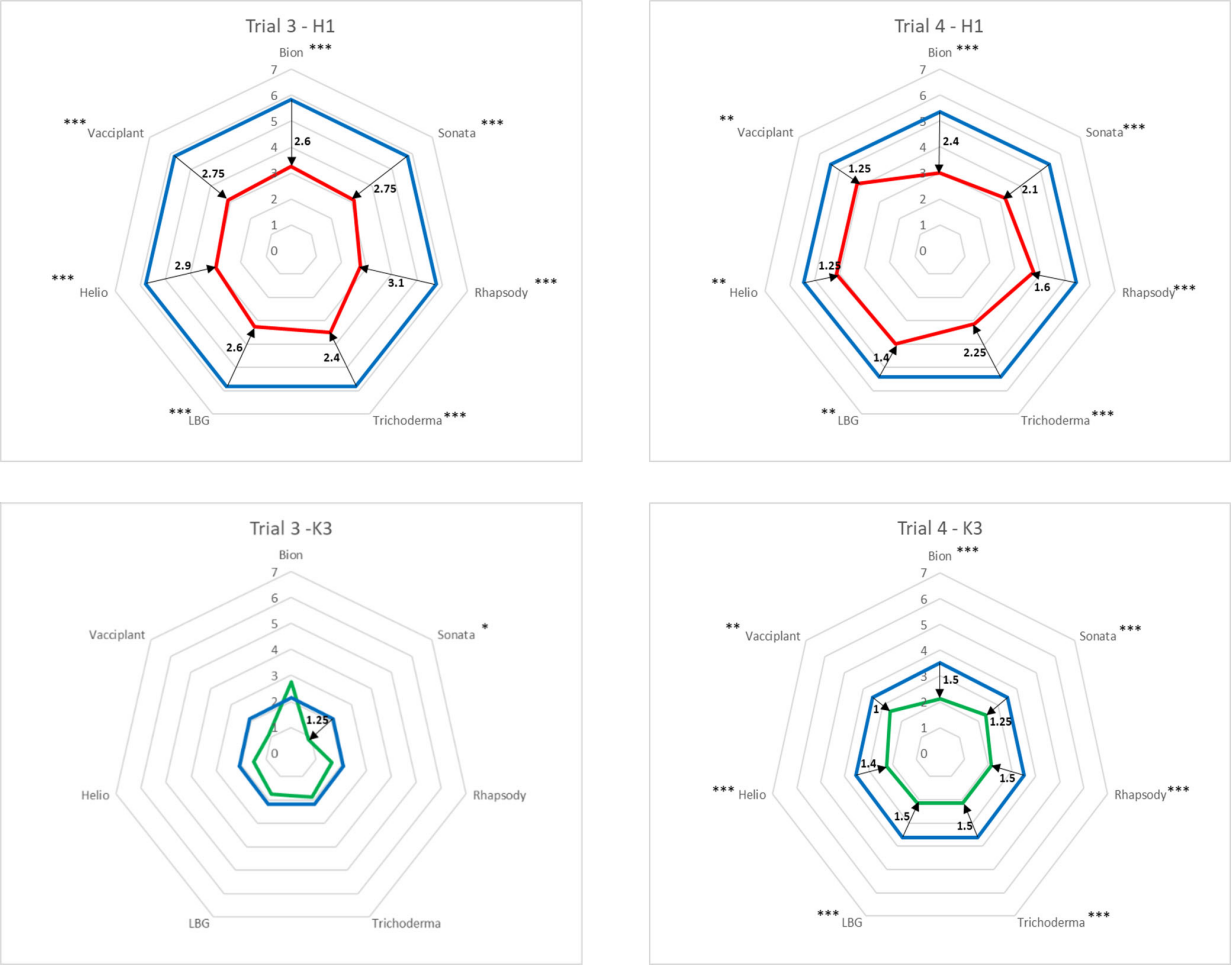
Protection obtained in Trials 3 and 4 with six bioPPPs and Bion^®^ (**Table 1**) against *Ad* on two carrot genotypes: H1 (red radars) and K3 (green radars). Radar units are the disease indexes (DIs) evaluated on a 0 (no symptom) to 9 (completely blighted plant) scale as described by Le Clerc et al. ^16^ Protection efficacy (black arrows and numbers) is represented by the difference of DI in treated modalities (red and green lines) and control modality (blue lines). These values are the differences in mean between products and water from the two scoring dates and the four replicates (estimate column in emmeans results in R, **Supplementary Material 1**). For each trial and each genotype, a pairwise comparison to water (Dunnett’s results, **Supplementary Material 1**) is given with the efficacy significance codes (*** <0.001, ** <0.01, and * < 0.05). bioPPPs, biological plant protection products.

In Trial 3, using the zero-to-nine scale to evaluate Ad symptoms, a minimal significant difference of 2.4 points was obtained for the susceptible H1 genotype treated with the various products compared to the water control. The most pronounced differences between the water control and the bioPPP treatments were observed for Sonata^®^, Rhapsody^®^, Helioterpen^®^ soufre, and Vacciplant^®^ (**Figure 5**; **Supplementary Material 1**). In Trial 4, the highest levels of protection were achieved with Sonata^®^, Trichoderma, and Bion^®^. However, regardless of the product, the protection observed in Trial 4 was lower than that in Trial 3, although reductions in symptom development remained significant. The largest difference in efficacy between the two trials was noted for Helioterpen^®^ soufre, which demonstrated a 2.9-point reduction in disease index relative to the control in Trial 3, compared to a 1.25-point reduction in Trial 4.

The expected difference in resistance was obtained between the two genotypes, with K3 being significantly more resistant than H1, as reflected in the radar chart sizes. Consequently, regardless of the bioPPP treatment, the overall protection conferred by treatments was lower in K3 compared to H1. In Trial 3, significant protection for K3 was achieved only with Sonata^®^ (**Figure 5**; **Supplementary Material 1**), whereas in Trial 4, all bioPPP treatments provided significant protection.

Considering together the two trials (3 and 4) and two genotypes (H1 and K3), the highest level of protection was achieved with Sonata^®^.

#### Stimulation of defence genes in the H1 genotype during Trial 4

**Figure 6:**
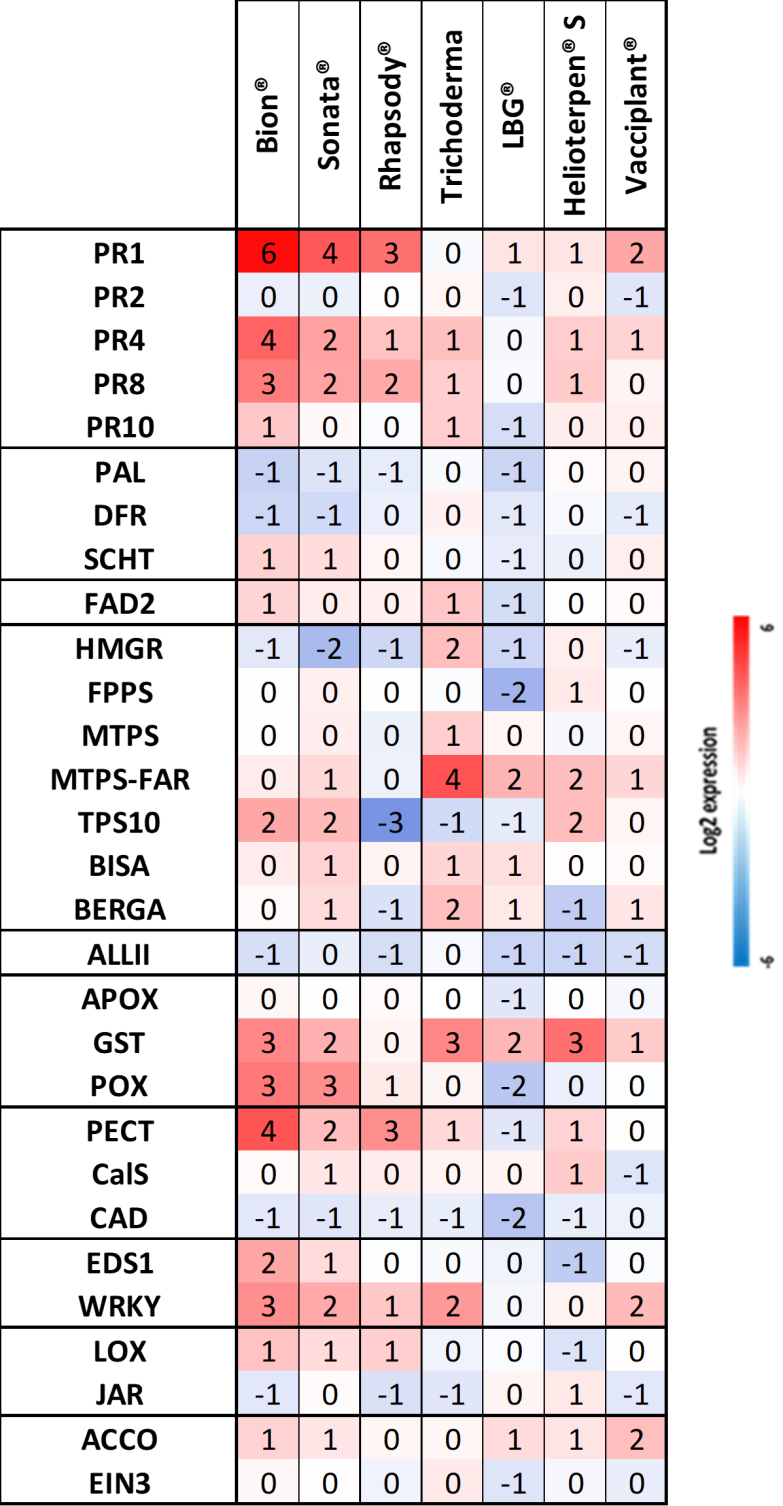
Relative defence gene expression in the susceptible genotype H1 sprayed with different bioPPPs and Bion^®^ in Trial 4. Helioterpen^®^ S, Helioterpen^®^ soufre; LBG^®^, LBG 01F34^®^. Each value is a pool of four biological replicates sampled 48 hpt and the mean of three technical replicates. The calibrator is the water. bioPPPs, biological plant protection products.

Treatments with Sonata^®^ and Bion^®^ resulted in substantial upregulation of six of the seven previously identified genes: *PR1*, *PR4*, *PR8*, *POX*, *PECT*, and *WRKY*. The seventh gene, *EDS1*, showed a lesser degree of induction (**Figure 6**). A similar gene expression pattern was observed with Rhapsody^®^, although with slightly lower modulation amplitudes. As noted above, Bion^®^, Sonata^®^, and Rhapsody^®^ were among the four products that conferred the highest protection against *Ad* in Trial 4 (**Figure 5**). Trichoderma, which also provided one of the highest levels of protection, exhibited a markedly different gene expression profile, with minimal effects on most genes except for *MTPS*-*FAR* and *GST* genes. For LBG 01F34^®^, which offered intermediate protection, no significant stimulation of defence genes was observed. Similarly, Helioterpen^®^ soufre and Vacciplant^®^, which also conferred intermediate protection, showed no strong induction of defence genes, apart from *GST* for Helioterpen^®^ soufre.

Regarding Helioterpen^®^ soufre, both protection efficacy and gene upregulation were lower in Trial 4 compared to Trial 3 (**Figures 4**–**6**).

#### Genotype receptivity to Sonata^®^

**Figure 7:**
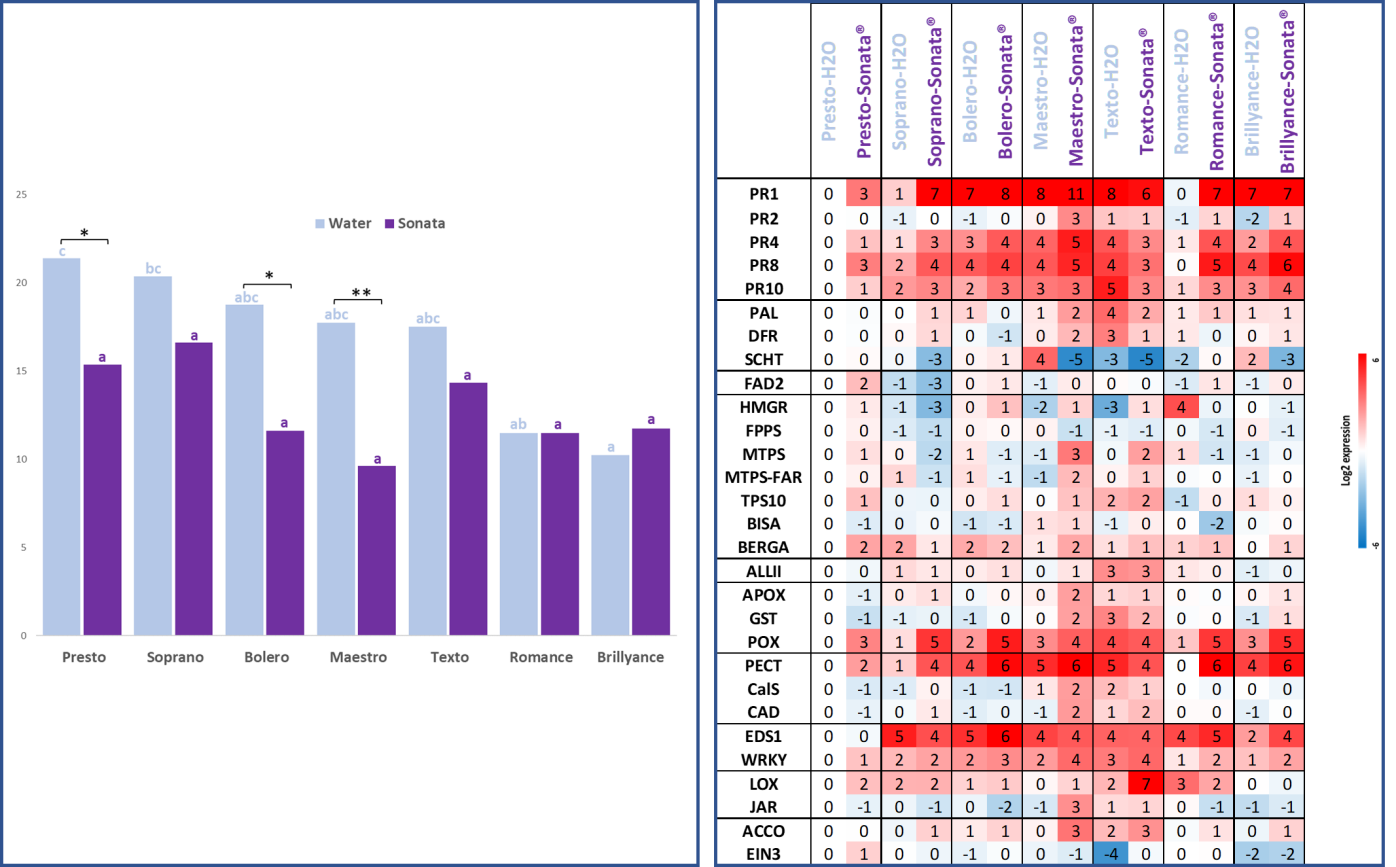
On the left, AUDPC mean scores were calculated from six scoring dates and four replicates for seven varieties treated with Sonata^®^ or water (control). The blue letters indicate Tukey’s groups for water and the violet ones for Sonata^®^. The star “*” indicates significant differences between treated and control modalities with a Tukey’s HSD (**Supplementary Material 2**). Test. Signif. Codes: ** <0.01 and * <0.05. On the right, relative defence gene expression 48 hours post-treatment with water or Sonata^®^ for the same varieties compared to the reference Presto treated with water. Overexpression is in red and underexpression in blue. Varieties are ordered from the most susceptible (Presto and Soprano) to the most resistant varieties (Romance and Brillyance). AUDPC, area under the disease progression curve; HSD, honestly significant difference.

As expected, differences in resistance among the panel of varieties were observed. This is evident in the untreated (water) control conditions (blue bars in **Figure 7**), where varying levels of disease severity were recorded, ranging from the highly susceptible varieties Presto and Soprano to the highly resistant Brillyance and Romance, with statistically significant differences (denoted by blue letters). The varieties Bolero, Maestro, and Texto displayed intermediate levels of resistance. The treatment effect was also highly significant (**Supplementary Material 2**), leading to a reduction in disease levels such that the differences between varieties were no longer significant after treatment (denoted by violet letters). When comparing each variety pairwise between the untreated control and Sonata^®^ treatment, statistically significant protection due to Sonata^®^ was observed for Maestro, Bolero, and Presto and not for the other varieties.

As also shown in **Figure 7**, under the water control condition, all resistant varieties except Romance exhibited overexpression of the seven genes *PR1*, *PR4*, *PR8*, *POX*, *PECT*, *EDS1*, and *WRKY* compared to Presto. Among the three varieties significantly protected by Sonata^®^ (Presto, Maestro, and Bolero), most of the seven genes were overexpressed in the Sonata^®^ treatment compared to the water control within the same variety, except *PR8* in Bolero and *EDS1* in Maestro and Presto. Additionally, Maestro exhibited upregulation of numerous other defence-related genes, particularly those involved in the isoprenoid, oxidative stress, and cell wall modification pathways. For Romance and Brillyance, which showed no protection following the Sonata^®^ treatment, nearly all seven genes were also overexpressed under the Sonata^®^ condition compared to the control, with the exception of the *PR1* gene in Brillyance. For the two varieties with slight but statistically non-significant protection after the Sonata^®^ treatment (Texto and Soprano), changes in gene expressions differed substantially. Soprano exhibited generally high induction of the seven genes, whereas in Texto, most of these genes were underexpressed after the Sonata^®^ treatment compared to the water control.

## Discussion

Although carrot growers are currently seeking effective biopesticides against Ad fungus, no product has yet been registered for this pathosystem, which poses a significant threat to crops. Encouraging pre-screening results with bioPPP candidates have been obtained. However, depending on whether the bioPPP acts as a PRI or a direct fungicide, whether the active ingredient is living or inert, thermosensitive or not, the associated management strategies will differ. To identify PRIs among biopesticides, we developed a biomolecular tool for monitoring the expression of carrot defence genes.

### Development and validation of a biomolecular tool for carrot

The tool was initially tested on the susceptible carrot genotype H1 following treatment with the well-characterised PRI, Bion^®^. After evaluating primer sequences for specificity and amplification efficiency, as well as assessing gene expression stability and reproducibility over time, we successfully established a tool that captures most pathways of the well-known plant defence mechanisms. This tool is now available under the reference DI-RV-19-0075 (extension of Patent n° WO/2011/161388; Brisset et al., 2019), along with a detailed methodology for its effective use.

To assess the reproducibility of this defence-monitoring tool, elicitation by Bion^®^ and Helioterpen^®^ soufre was evaluated across multiple independent greenhouse trials. While reproducibility was consistently high for Bion^®^ treatment across all trials, and similarly high for Helioterpen^®^ soufre during the first three trials, a notably poor induction with almost no elicitation of key defence genes was observed for Helioterpen^®^ soufre in Trial 4. Interestingly, in Trial 4, a much lower level of protection was also achieved with this product. Therefore, it is likely not the reproducibility of the molecular tool itself that is in question but rather the influence of experimental conditions. Trials 3 and 4 were conducted in the same greenhouse using identical growing practices. However, while Trial 3 was sown on March 11, Trial 4 was sown 22 days later, on April 1. The temperature during bioPPP application was higher in Trial 4 compared to Trial 3, which may have contributed to the reduced efficacy of Helioterpen^®^ soufre. The manufacturer advises against using Helioterpen^®^ soufre when temperatures exceed 28°C–30°C under cover for 24 hours. In Trial 4, maximum temperatures reached approximately 28°C for three consecutive days after spraying, potentially explaining the lower level of protection observed. Although light quality was not assessed in either trial, unavoidable differences could also have contributed to the observed variations in the results as reported in the literature (Hellström et al., 2022; Yu and Lee, 2013). We hypothesise that Helioterpen^®^ soufre may be significantly more sensitive to these variations in experimental conditions compared to Bion^®^. Further studies are needed to assess the effects of temperature and light condition fluctuations on Helioterpen^®^ soufre.

### Identification of the plant resistance inducers for carrot protection against *Ad*

Statistically efficient protection was observed with all products in Trial 3 on the susceptible genotype H1, with a minimum 2-point decrease on the rating scale. These findings are consistent with previous results (Moussa et al., 2024). 

Similar protection and gene expression profile patterns were observed for Bion^®^ and Sonata^®^. While Bion^®^ is a well-known inducer of plant defences in many species, with no direct action on the pathogen, Sonata^®^, which contains *Bacillus pumilus* strain QST 2808, is primarily reported as a biological fungicide capable of preventing powdery mildew in grapes. The bacteria produce an antifungal amino sugar compound that disrupts the pathogen’s cell wall (Ortiz and Sansinenea, 2021). Our results highlight that Sonata^®^ also clearly acts as a plant resistance inducer in carrots against Ad. However, a fungicidal or fungistatic effect of Sonata^®^ cannot be excluded, as this has not yet been tested on the fungus.

Among the 29 defence genes included in the biomolecular tool, seven were particularly elicited by Bion^®^ and Helioterpen^®^ soufre: *PR1*, *PR4*, *PR8*, *POX*, *PECT*, *EDS1*, and *WRKY*. The two products, Sonata^®^ and Bion^®^, were able to activate the systemic acquired resistance (SAR) pathway as evidenced by the overexpression of *PR* protein genes. PR proteins can act directly on the pathogen cell walls through hydrolytic activity or other antimicrobial mechanisms or indirectly through enzymes that release elicitors (Hammerschmidt, 1999). They are described in the literature as the primary elements activated early following a pathogen attack. In Wally and Punja (2010) demonstrated that the peroxidase overexpression in transgenic carrot plants, which conferred resistance to necrotrophic pathogens, was linked to increased *PR* transcript accumulation and the rapid removal of H_2_O_2_ during the oxidative burst response. Among all the elicited genes, *PR1* was one of the most overexpressed following treatment. PR1 proteins are known to be the most abundantly produced pathogenesis-related proteins during a pathogen attack. However, their *in vivo* function remains unclear, as they may have multiple roles, including antimicrobial function, defence signal amplification, sterol recognition, or effector recognition (Breen et al., 2017). *PR4* has also been reported to exhibit antifungal activity in various plant species. Bai et al. (2013) suggested that the widespread expression pattern of the *MdPR4* gene, which encodes a PR protein involved in the defence response of *Malus domestica* against *Botryosphaeria dothidea*, could be attributed to the diverse physiological functions of *PR4* genes. Furthermore, they found that *MdPR4* gene expression was regulated by salicylic acid (SA) and jasmonic acid (JA) signalling pathways.

In the oxidative stress pathway, the overexpression of the *POX* gene observed in our study is consistent with the findings of Wally et al. (2009), who reported that peroxidase overexpression in transgenic carrot plants conferred resistance to two foliar necrotrophic pathogens, *Sclerotinia sclerotiorum* and *B. cinerea*.

The *PECT* gene was also highly upregulated by the two products. This gene encodes pectin methylesterase (PME), an enzyme that, depending on the cell wall properties, can either strengthen or weaken the cell wall (Wattier, 2013). Overexpression of the *PECT* gene may indicate a loss of cell wall integrity, leading to the formation of signalling molecules, as described by Wormit and Usadel (2018). Messiaen and Van Cutsem (1993) previously demonstrated that certain pectic fragments can induce defence gene expression in carrot cell suspensions. Boedo et al. (2010) reported that the penetration of carrot leaves by Ad fungus was more difficult in a resistant cultivar than in a susceptible one. In this context, PME activity may contribute to resistance by altering the pattern of pectin methyl esterification in the cell wall, as observed by Bethke et al. (2014) in the interaction between *Arabidopsis* and *Pseudomonas syringae*.

The two treatments also led to an overexpression of *EDS1* and *WRKY*, genes associated with the SA pathway. The *EDS1* gene encodes a lipase-like protein that serves as a transcriptional coactivator essential for transcriptomic reprogramming during SA-dependent plant immunity (Khan et al., 2022). The *WRKY* transcription factor may enhance resistance by upregulating SA-responsive genes, as demonstrated in the *Arabidopsis*/*P. syringae* pathosystem (Hu et al., 2012). According to the literature, the SA signalling pathway is predominantly implicated in the control of biotrophic pathogens, whereas JA is more effective against necrotrophic or hemi-biotrophic pathogens. Given that Ad is reported to be a necrotrophic fungus, activation of the SA pathway following these treatments may not intuitively seem the most effective strategy against this pathogen. Nevertheless, the observed crop protection after treatment and fungal inoculation was highly effective.

A similar gene expression pattern, albeit with lower intensity, was observed following the Rhapsody^®^ treatment. This reduced elicitation may account for the lower level of protection observed in Trial 4 with this bioPPP compared to the two other products.

Trichoderma, which provides highly significant protection to H1 in Trial 4, induces very low or no upregulation of most defence genes, except for *GST* and *MTPS-FAR* genes. Gullner et al. (2018) reported that the expression of multiple *GST*s is activated via the SA pathway and that plants treated with beneficial bacteria or fungi can induce systemic resistance response through the upregulation of *GST* genes. Similar to POX proteins, GST proteins are capable of mitigating oxidative stress and detoxifying harmful substances by conjugating them with glutathione. The *MTPS-FAR* gene, which encodes enzymes such as monoterpene synthase (MTPS), may also play a role in carrot defence. Previous studies have identified that the accumulation of certain terpenes contributes to carrot resistance to Ad, with resistant genotypes accumulating higher levels of specific terpene compounds compared to the susceptible ones (Koutouan et al., 2018). Moreover, resistance and metabolic quantitative trait loci (QTLs) were found to colocalise on the carrot genome, and these terpenes exhibited direct toxicity on the fungus by significantly inhibiting mycelial growth (Koutouan et al., 2023). Thus, the significant overexpression of only two genes, *GST* and *MTPS-FAR*, may be sufficient to enhance plant protection. Alternatively, Trichoderma may confer protection against Ad primarily through competitive interactions, as demonstrated in other pathosystems (Sood et al., 2020), rather than through a mechanism based on defence gene elicitation.

For the other bioPPPs, Vacciplant^®^ and LBG 01F34^®^, although less favourable experimental conditions—similar to those suggested for Helioterpen^®^ soufre or Rhapsody^®^—cannot be entirely ruled out, we hypothesise that the observed protection was likely primarily due to a direct effect on the fungus, as no significant elicitation of defence genes was detected.

In summary, some bioPPPs can be classified as PRIs on carrot, while some others likely do not act as PRIs and instead provide protection through direct effects on the fungus or through alternative, as-yet-unidentified pathways. Toxicity assays will be necessary to distinguish between the contributions of induced resistance and any direct antifungal effects of the products. Furthermore, an association of direct and indirect mechanisms is possible and has been frequently reported for PRIs. Understanding the mode of action of each bioPPP will be critical for designing effective combinations of these products in integrated production systems.

### Carrot genotype receptivity to Sonata^®^

Three distinct protection profiles were observed following the Sonata^®^ treatment: no protection (Romance and Brillyance), strong protection (Presto, Maestro, and Bolero), or a slight but non-significant protective effect (Texto and Soprano). With the exception of Texto, where defence genes were underexpressed after Sonata^®^ application, all six other varieties were receptive to the treatment.

The lack of efficacy observed in the two most resistant varieties, Brillyance and Romance, is likely due to their already high constitutive levels of resistance. In Brillyance, this hypothesis is supported by the high constitutive expression of defence genes observed with qPFD chip (Brillyance-water). In Romance, however, the constitutive expression of defence genes was low. This suggests that resistance mechanisms may be mediated by other genes not included on the chip. Despite this, Romance defence genes were induced by the Sonata^®^ treatment, indicating that induced resistance is not sufficient to enhance pre-existing resistance mechanisms in this variety.

The strong protective effect conferred by the Sonata^®^ treatment in Maestro, Bolero, and Presto—representing two intermediate resistant and one very susceptible genotype, respectively—was strongly correlated with the induction of numerous defence genes. Although the constitutive level of defence gene expression is the lowest in Presto, the Sonata^®^ treatment was sufficiently effective to provide a noteworthy level of protection. It would be valuable to investigate whether two or three additional sprays would further enhance defence responses in Presto or whether a combination with other bioPPPs would be beneficial.

The third profile corresponds to the slight, but not significant, protective effect of Sonata^®^ observed in Soprano and Texto. For Texto, the gene expression of numerous genes previously identified was already constitutively high, and the PRI was unable to stimulate them, instead tending to repress their expression. The modest protective effect observed may be due to a slight enhancement of genes involved in the isoprenoid pathway, which has been identified as crucial for carrot resistance to Ad (Koutouan et al., 2023). In the case of Soprano, although global gene expression was high following the Sonata^®^ treatment, a significant number of genes in the isoprenoid pathway were underexpressed. In this instance, the positive effect of Sonata^®^ on the activation of early defence pathways, through the elicitation of PR genes, may have been counterbalanced by its negative impact on the isoprenoid pathway, which is also known to play an important role in carrot resistance to Ad (Koutouan et al., 2018).

The study highlighted that the receptivity of the bioPPP was clearly dependent on the variety, as different levels of protection were observed following the Sonata^®^ treatment across the seven varieties. The genotype effect involves two sequential mechanisms: 1) sensitivity to the PRI, which may or may not result in the induction of defence genes, and 2) the enhancement of resistance, or lack thereof. Carrot sensitivity to PRI does not correlate with the constitutive level of resistance in the varieties.

## Future insights

Our newly developed efficient biomolecular tool for carrot will enable the screening of other PRIs among bioPPPs for carrot protection against Ad. It will facilitate the evaluation of environmental impacts on PRI efficacy and allow assessment of whether multiple applications may enhance defence gene expression, potentially leading to improved protection. Additionally, this tool could assist breeders in selecting carrot varieties that are receptive to PRIs. To this end, further analyses on a broader panel of carrot varieties are underway to identify genetic factors underlying these interaction specificities. In the medium to long term, plant receptivity to PRI could become a novel breeding trait to consider. When combined with constitutive resistance factors, this approach could contribute to the development of durable resistant varieties for producers.

Experimental studies are essential to identify the variety–product pairs that demonstrate optimal performance. As part of the European program H2020 called OPTIMA, initial field trials conducted under diverse environmental conditions in France and Greece have shown promising results in terms of protection across different varieties. This information will support extension services in developing guidelines for the optimal variety–bioPPP combinations and best practices for their application under field conditions. For example, based on current findings with Helioterpen^®^ soufre, growers are advised to apply this product either very early in the morning or late in the evening during hot weather conditions. Various other environmental factors can influence the effectiveness of PRIs, including soil properties, nitrogen fertilisation, and soil organic matter content (Reglinski et al., 2023). Temperature and humidity fluctuations can significantly reduce their shelf life, thereby limiting field efficacy (Kumar et al., 2021). As a result, repeated applications may be required. Biopesticides are generally considered to have a lower environmental impact than synthetic pesticides due to their specific modes of action, reduced persistence in the soil environment (Ayilara et al., 2023), absence of problematic residues, and rapid decomposition. If these attributes are confirmed for bioPPPs effective in carrot protection, repeated applications may indeed be advised. Additionally, diverse modes of action identified among the seven bioPPPs studied suggest the potential for complementary benefits when strategically combined in an integrated pest management program.

In conclusion, bioPPPs show promise as potential replacements for synthetic products. However, their widespread adoption remains challenging and will require further research to fully harness their potential.

## Data Availability

Original datasets are available in a publicly accessible repository: The original contributions presented in the study are publicly available. This data can be found here: https://doi.org/10.57745/0XRFJC.
