# Supplementary material for: Identification of plant resistance inducers and evaluation of genotype receptivity for carrot protection against Alternaria leaf blight
Source: Front Plant Sci. 2025 Mar 5;16:1513301. doi: 10.3389/fpls.2025.1513301 (PMC11921781; doi:10.3389/fpls.2025.1513301)
Supplement: Supplementary file 1 [file DataSheet1.docx]

Supplementary Material 2

Statistics of trial 5 : AUDPC of all the dates

Table of contents

[For water and all the varieties, AUDPC value per repetition (tunnel) 1](#_Toc170244870)

[For Sonata and all the varieties, AUDPC value per repetition (tunnel) 2](#_Toc170244871)

[ANOVA model on AUDPC values 4](#_Toc170244872)

[Postulates verification 4](#_Toc170244873)

[Pairwise comparison between varieties 5](#_Toc170244874)

[Pairwise comparison between modalities (water and sonata) 7](#_Toc170244875)

# For water and all the varieties, AUDPC value per repetition (tunnel)

varieties=unique(df4$variete)
AUDPC_values <- list()
for (variety in varieties) {
 AUDPC_values[[variety]] <- list()

 for (tunnel_number in 1:4) {
 evaluation <- df4$score[df4$variete == variety & df4$tunnel == tunnel_number]
 dates <- as.numeric(df4$time[df4$variete == variety & df4$tunnel == tunnel_number])
 AUDPC <- audpc(evaluation, dates, type = "absolute")
 AUDPC_values[[variety]][[tunnel_number]] <- AUDPC
 }
}

results <- data.frame(variete = character(), AUDPC = numeric(), stringsAsFactors = FALSE)

# Populate the data.frame with AUDPC values
for (variety in varieties) {
 for (tunnel_number in 1:4) {
 results <- rbind(results, data.frame(variete = variety, AUDPC = AUDPC_values[[variety]][[tunnel_number]], stringsAsFactors = FALSE))
 }
}
results

variete AUDPC
evaluation Presto 30.0
evaluation1 Presto 20.5
evaluation2 Presto 18.0
evaluation3 Presto 17.0
evaluation4 Boléro 18.5
evaluation5 Boléro 16.0
evaluation6 Boléro 25.0
evaluation7 Boléro 15.5
evaluation8 Soprano 19.5
evaluation9 Soprano 19.0
evaluation10 Soprano 22.0
evaluation11 Soprano 21.0
evaluation12 Texto 15.5
evaluation13 Texto 17.0
evaluation14 Texto 18.0
evaluation15 Texto 19.5
evaluation16 Romance 7.0
evaluation17 Romance 13.5
evaluation18 Romance 13.0
evaluation19 Romance 12.5
evaluation20 Brillyance 7.5
evaluation21 Brillyance 9.5
evaluation22 Brillyance 17.5
evaluation23 Brillyance 6.5
evaluation24 Maestro 21.0
evaluation25 Maestro 24.5
evaluation26 Maestro 15.0
evaluation27 Maestro 10.5

res1=data.frame(results, modalite="Water")

# For Sonata and all the varieties, AUDPC value per repetition (tunnel)

df5=subset(df3, df3$modalite=="Sonata")
df5

# A tibble: 162 × 8
 variete tablette tunnel modalite id2 id time score
 <fct> <dbl> <fct> <fct> <chr> <fct> <fct> <dbl>
 1 Presto 2 1 Sonata ind8 ind4 Date1 0
 2 Boléro 2 1 Sonata ind9 ind1 Date1 0
 3 Soprano 2 1 Sonata ind10 ind6 Date1 2
 4 Texto 2 1 Sonata ind11 ind7 Date1 1
 5 Romance 2 1 Sonata ind12 ind5 Date1 0
 6 Brillyance 2 1 Sonata ind13 ind2 Date1 0
 7 Maestro 2 1 Sonata ind14 ind3 Date1 0
 8 Presto 3 2 Sonata ind22 ind11 Date1 0
 9 Boléro 3 2 Sonata ind23 ind8 Date1 1
10 Soprano 3 2 Sonata ind24 ind13 Date1 0
# ℹ 152 more rows

varieties=unique(df5$variete)
#df5$varieties=droplevels(df5$varieties)
AUDPC_values <- list()
for (variety in varieties) {
 AUDPC_values[[variety]] <- list()

 # Texto has a missing value on tunnel 3
 if (variety == "Texto") {
 tunnel_range <- c(1,2,4)
 } else {
 tunnel_range <- 1:4
 }


 # Loop over tunnel numbers from 1 to 4
 for (tunnel_number in tunnel_range) {
 evaluation <- df5$score[df5$variete == variety & df5$tunnel == tunnel_number]
 dates <- as.numeric(df5$time[df5$variete == variety & df5$tunnel == tunnel_number])
 AUDPC <- audpc(evaluation, dates, type = "absolute")
 AUDPC_values[[variety]][[tunnel_number]] <- AUDPC
 }
}

AUDPC_values$Texto[[3]]=NA
# Create a data.frame to store the results
results <- data.frame(variete = character(), AUDPC = numeric(), stringsAsFactors = FALSE)

# Populate the data.frame with AUDPC values

for (variety in varieties) {

 tunnel_range <- 1:4

 for (tunnel_number in tunnel_range) {
 results <- rbind(results, data.frame(variete = variety, AUDPC = AUDPC_values[[variety]][[tunnel_number]], stringsAsFactors = FALSE))
 }
}

View(results)

res2=data.frame(results, modalite="Sonata")

Tab_AUDPC=rbind(res1,res2)

# ANOVA model on AUDPC values

 mod.complet=lm(Tab_AUDPC$AUDPC~variete*modalite, data=Tab_AUDPC)
Anova(mod.complet, test.statistic = "F")

Anova Table (Type II tests)

Response: Tab_AUDPC$AUDPC
 Sum Sq Df F value Pr(>F)
variete 433.33 6 4.1835 0.002269 **
modalite 200.43 1 11.6102 0.001481 **
variete:modalite 154.95 6 1.4959 0.203728
Residuals 707.79 41
---
Signif. codes: 0 '***' 0.001 '**' 0.01 '*' 0.05 '.' 0.1 ' ' 1

### Postulates verification

shapiro.test(residuals(mod.complet))

Shapiro-Wilk normality test

data: residuals(mod.complet)
W = 0.99019, p-value = 0.9329

plot(residuals(mod.complet) ~ fitted(mod.complet))
abline(h = 0, col = "red")


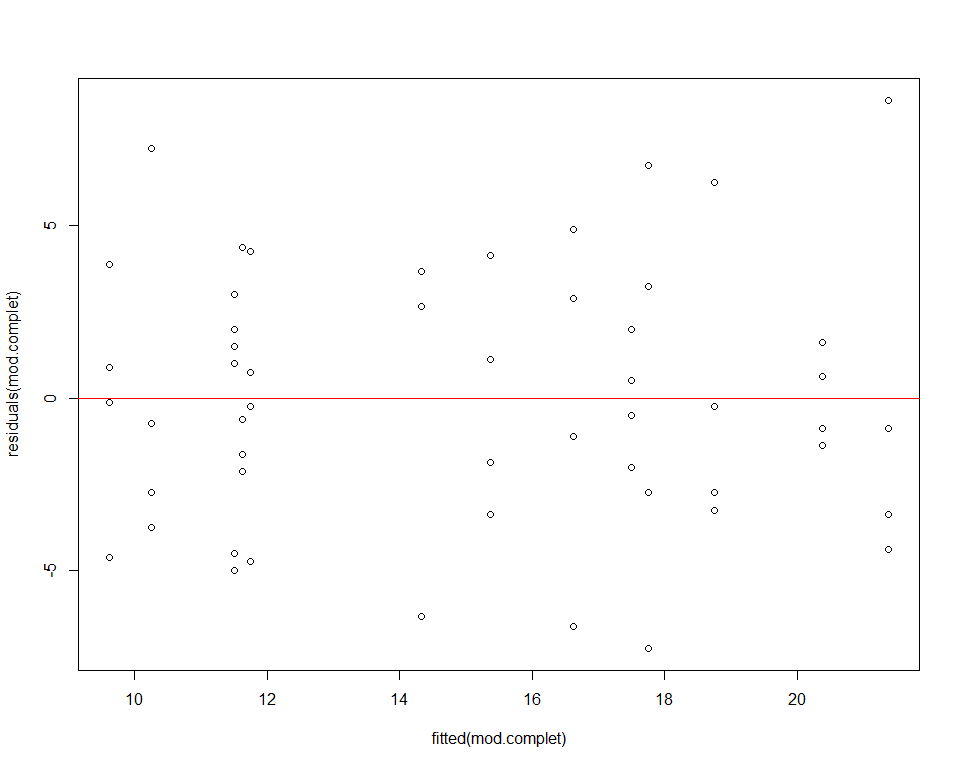


## Pairwise comparison between varieties

emm_var<- emmeans(mod.complet, pairwise ~variete | modalite)
emm_var$contrasts

modalite = Water:
 contrast estimate SE df t.ratio p.value
 Presto - Maestro 3.625 2.94 41 1.234 0.8767
 Presto - Boléro 2.625 2.94 41 0.893 0.9715
 Presto - Romance 9.875 2.94 41 3.361 0.0259
 Presto - Brillyance 11.125 2.94 41 3.787 0.0082
 Presto - Texto 3.875 2.94 41 1.319 0.8392
 Presto - Soprano 1.000 2.94 41 0.340 0.9999
 Maestro - Boléro -1.000 2.94 41 -0.340 0.9999
 Maestro - Romance 6.250 2.94 41 2.127 0.3566
 Maestro - Brillyance 7.500 2.94 41 2.553 0.1677
 Maestro - Texto 0.250 2.94 41 0.085 1.0000
 Maestro - Soprano -2.625 2.94 41 -0.893 0.9715
 Boléro - Romance 7.250 2.94 41 2.468 0.1979
 Boléro - Brillyance 8.500 2.94 41 2.893 0.0811
 Boléro - Texto 1.250 2.94 41 0.425 0.9995
 Boléro - Soprano -1.625 2.94 41 -0.553 0.9977
 Romance - Brillyance 1.250 2.94 41 0.425 0.9995
 Romance - Texto -6.000 2.94 41 -2.042 0.4051
 Romance - Soprano -8.875 2.94 41 -3.021 0.0603
 Brillyance - Texto -7.250 2.94 41 -2.468 0.1979
 Brillyance - Soprano -10.125 2.94 41 -3.446 0.0208
 Texto - Soprano -2.875 2.94 41 -0.979 0.9559

modalite = Sonata:
 contrast estimate SE df t.ratio p.value
 Presto - Maestro 5.750 2.94 41 1.957 0.4563
 Presto - Boléro 3.750 2.94 41 1.276 0.8586
 Presto - Romance 3.875 2.94 41 1.319 0.8392
 Presto - Brillyance 3.625 2.94 41 1.234 0.8767
 Presto - Texto 1.042 3.17 41 0.328 0.9999
 Presto - Soprano -1.250 2.94 41 -0.425 0.9995
 Maestro - Boléro -2.000 2.94 41 -0.681 0.9930
 Maestro - Romance -1.875 2.94 41 -0.638 0.9951
 Maestro - Brillyance -2.125 2.94 41 -0.723 0.9903
 Maestro - Texto -4.708 3.17 41 -1.484 0.7526
 Maestro - Soprano -7.000 2.94 41 -2.383 0.2319
 Boléro - Romance 0.125 2.94 41 0.043 1.0000
 Boléro - Brillyance -0.125 2.94 41 -0.043 1.0000
 Boléro - Texto -2.708 3.17 41 -0.853 0.9773
 Boléro - Soprano -5.000 2.94 41 -1.702 0.6187
 Romance - Brillyance -0.250 2.94 41 -0.085 1.0000
 Romance - Texto -2.833 3.17 41 -0.893 0.9716
 Romance - Soprano -5.125 2.94 41 -1.744 0.5914
 Brillyance - Texto -2.583 3.17 41 -0.814 0.9821
 Brillyance - Soprano -4.875 2.94 41 -1.659 0.6458
 Texto - Soprano -2.292 3.17 41 -0.722 0.9904

P value adjustment: tukey method for comparing a family of 7 estimates

cld_results <- cld(emm_var, Letters = letters, adjust = "tukey")

cld_results

modalite = Water:
 variete emmean SE df lower.CL upper.CL .group
 Brillyance 10.25 2.08 41 4.38 16.1 a
 Romance 11.50 2.08 41 5.63 17.4 ab
 Texto 17.50 2.08 41 11.63 23.4 abc
 Maestro 17.75 2.08 41 11.88 23.6 abc
 Boléro 18.75 2.08 41 12.88 24.6 abc
 Soprano 20.38 2.08 41 14.51 26.2 bc
 Presto 21.38 2.08 41 15.51 27.2 c

modalite = Sonata:
 variete emmean SE df lower.CL upper.CL .group
 Maestro 9.62 2.08 41 3.76 15.5 a
 Romance 11.50 2.08 41 5.63 17.4 a
 Boléro 11.62 2.08 41 5.76 17.5 a
 Brillyance 11.75 2.08 41 5.88 17.6 a
 Texto 14.33 2.40 41 7.56 21.1 a
 Presto 15.38 2.08 41 9.51 21.2 a
 Soprano 16.62 2.08 41 10.76 22.5 a

Confidence level used: 0.95

Conf-level adjustment: sidak method for 7 estimates

P value adjustment: tukey method for comparing a family of 7 estimates

significance level used: alpha = 0.05

## Pairwise comparison between modalities (water and sonata)

emm_mod<- emmeans(mod.complet, pairwise ~modalite | variete)
emm_mod$contrasts

variete = Presto:
 contrast estimate SE df t.ratio p.value
 Water - Sonata 6.00 2.94 41 2.042 0.0476

variete = Maestro:
 contrast estimate SE df t.ratio p.value
 Water - Sonata 8.12 2.94 41 2.766 0.0085

variete = Boléro:
 contrast estimate SE df t.ratio p.value
 Water - Sonata 7.12 2.94 41 2.425 0.0198

variete = Romance:
 contrast estimate SE df t.ratio p.value
 Water - Sonata 0.00 2.94 41 0.000 1.0000

variete = Brillyance:
 contrast estimate SE df t.ratio p.value
 Water - Sonata -1.50 2.94 41 -0.511 0.6124

variete = Texto:
 contrast estimate SE df t.ratio p.value
 Water - Sonata 3.17 3.17 41 0.998 0.3242

variete = Soprano:
 contrast estimate SE df t.ratio p.value
 Water - Sonata 3.75 2.94 41 1.276 0.2090

cld_results <- cld(emm_mod, Letters = letters, adjust = "tukey")

cld_results

variete = Presto:
 modalite emmean SE df lower.CL upper.CL .group
 Sonata 15.38 2.08 41 10.55 20.2 a
 Water 21.38 2.08 41 16.55 26.2 b

variete = Maestro:
 modalite emmean SE df lower.CL upper.CL .group
 Sonata 9.62 2.08 41 4.80 14.4 a
 Water 17.75 2.08 41 12.93 22.6 b

variete = Boléro:
 modalite emmean SE df lower.CL upper.CL .group
 Sonata 11.62 2.08 41 6.80 16.4 a
 Water 18.75 2.08 41 13.93 23.6 b

variete = Romance:
 modalite emmean SE df lower.CL upper.CL .group
 Water 11.50 2.08 41 6.68 16.3 a
 Sonata 11.50 2.08 41 6.68 16.3 a

variete = Brillyance:
 modalite emmean SE df lower.CL upper.CL .group
 Water 10.25 2.08 41 5.43 15.1 a
 Sonata 11.75 2.08 41 6.93 16.6 a

variete = Texto:
 modalite emmean SE df lower.CL upper.CL .group
 Sonata 14.33 2.40 41 8.76 19.9 a
 Water 17.50 2.08 41 12.68 22.3 a

variete = Soprano:
 modalite emmean SE df lower.CL upper.CL .group
 Sonata 16.62 2.08 41 11.80 21.4 a
 Water 20.38 2.08 41 15.55 25.2 a

Confidence level used: 0.95

Conf-level adjustment: sidak method for 2 estimates

significance level used: alpha = 0.05
