# Supplementary material for: Identification of plant resistance inducers and evaluation of genotype receptivity for carrot protection against Alternaria leaf blight
Source: Front Plant Sci. 2025 Mar 5;16:1513301. doi: 10.3389/fpls.2025.1513301 (PMC11921781; doi:10.3389/fpls.2025.1513301)
Supplement: Supplementary file 2 [file DataSheet2.docx]

Supplementary Material 1

Statistics of Trials 3 and 4 results

Table of contents

[Trial 3: genotype H1 1](#_Toc170242561)

[Model 1](#_Toc170242562)

[Postulates verification 2](#_Toc170242563)

[Pairwise comparisons (Tukey) 3](#_Toc170242564)

[Pairwise comparisons to water (Dunnett) 4](#_Toc170242565)

[Trial 3: genotype K3 5](#_Toc170242566)

[Model: 5](#_Toc170242567)

[Postulates verification 6](#_Toc170242568)

[Pairwise comparison (Tukey) 6](#_Toc170242569)

[Pairwise comparison to water (Dunnett) 7](#_Toc170242570)

[Trial 4: genotype H1 8](#_Toc170242571)

[Model: 8](#_Toc170242572)

[Postulates verification 9](#_Toc170242573)

[Pairwise comparisons (Tukey) 10](#_Toc170242574)

[Pairwise comparisons to water (Dunnett) 11](#_Toc170242575)

[Trial 4: genotype K3 12](#_Toc170242576)

[Model 12](#_Toc170242577)

[Postulates verification 12](#_Toc170242578)

[Pairwise comparisons (Tukey) 13](#_Toc170242579)

[Pairwise comparisons (Dunnett) 14](#_Toc170242580)

# Trial 3: genotype H1

## Model

res.lmer<- lmer(Score~product*time+(1|id)+(1|repetition), data=genH1)
Anova(res.lmer, test.statistic = "F")

Analysis of Deviance Table (Type II Wald F tests with Kenward-Roger df)

Response: Score
 F Df Df.res Pr(>F)
product 5.2534 7 21 0.00140 **
time 177.9177 1 24 1.358e-12 ***
product:time 2.9395 7 24 0.02264 *
---
Signif. codes: 0 '***' 0.001 '**' 0.01 '*' 0.05 '.' 0.1 ' ' 1

ranova(res.lmer)

ANOVA-like table for random-effects: Single term deletions

Model:
Score ~ product + time + (1 | id) + (1 | repetition) + product:time
 npar logLik AIC LRT Df Pr(>Chisq)
<none> 19 -74.954 187.91
(1 | id) 18 -78.955 193.91 8.0015 1 0.004674 **
(1 | repetition) 18 -75.131 186.26 0.3539 1 0.551897
---
Signif. codes: 0 '***' 0.001 '**' 0.01 '*' 0.05 '.' 0.1 ' ' 1

res.lmer<- lmer(Score~product+time+(1|id)+(1|repetition), data=genH1)
Anova(res.lmer, test.statistic = "F")

Analysis of Deviance Table (Type II Wald F tests with Kenward-Roger df)

Response: Score
 F Df Df.res Pr(>F)
product 5.2533 7 21 0.0014 **
time 123.7294 1 31 2.376e-12 ***
---
Signif. codes: 0 '***' 0.001 '**' 0.01 '*' 0.05 '.' 0.1 ' ' 1

ranova(res.lmer)

ANOVA-like table for random-effects: Single term deletions

Model:
Score ~ product + time + (1 | id) + (1 | repetition)
 npar logLik AIC LRT Df Pr(>Chisq)
<none> 12 -86.279 196.56
(1 | id) 11 -88.606 199.21 4.6545 1 0.03097 *
(1 | repetition) 11 -86.456 194.91 0.3539 1 0.55190
---
Signif. codes: 0 '***' 0.001 '**' 0.01 '*' 0.05 '.' 0.1 ' ' 1

### Postulates verification

shapiro.test(residuals(res.lmer))

Shapiro-Wilk normality test

data: residuals(res.lmer)
W = 0.96937, p-value = 0.1121


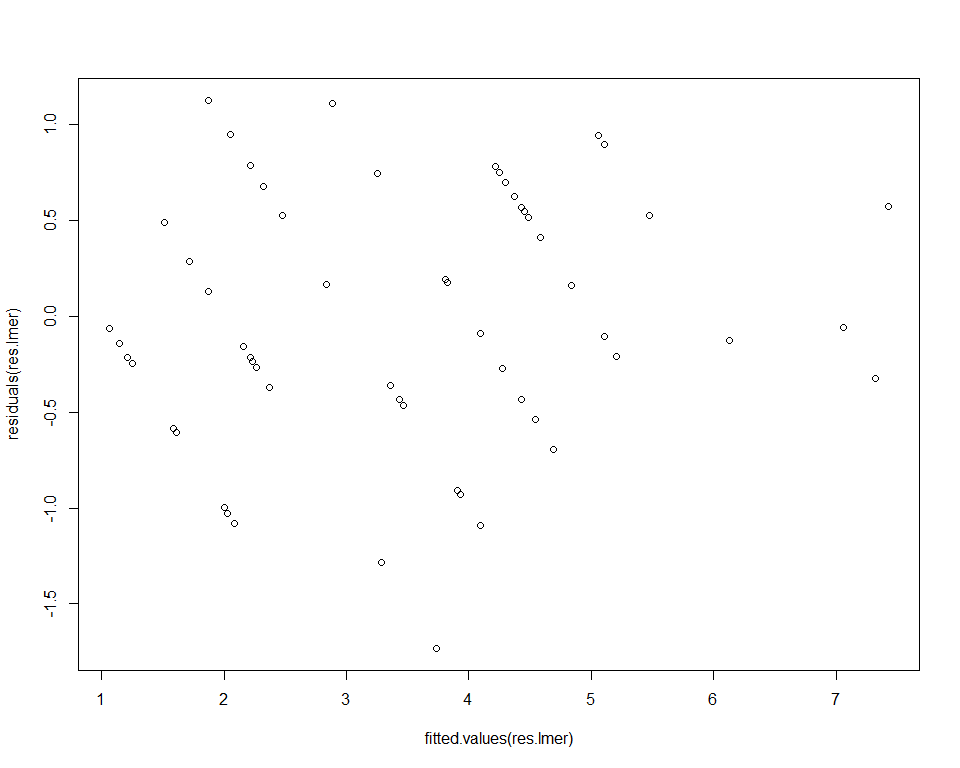


## Pairwise comparisons (Tukey)

emmeans(res.lmer, list(pairwise ~ product), adjust = "tukey")

$`emmeans of product`
 product emmean SE df lower.CL upper.CL
 Water 5.88 0.449 23.1 4.95 6.80
 Bion 3.25 0.449 23.1 2.32 4.18
 Helioterpene 3.00 0.449 23.1 2.07 3.93
 LBG 3.25 0.449 23.1 2.32 4.18
 Rhapsody 2.75 0.449 23.1 1.82 3.68
 Sonata 3.12 0.449 23.1 2.20 4.05
 Trichoderma 3.50 0.449 23.1 2.57 4.43
 Vacciplant 3.12 0.449 23.1 2.20 4.05

Results are averaged over the levels of: time
Degrees-of-freedom method: kenward-roger
Confidence level used: 0.95

$`pairwise differences of product`
 1 estimate SE df t.ratio p.value
 Water - Bion 2.625 0.611 21 4.298 0.0064
 Water - Helioterpene 2.875 0.611 21 4.708 0.0025
 Water - LBG 2.625 0.611 21 4.298 0.0064
 Water - Rhapsody 3.125 0.611 21 5.117 0.0010
 Water - Sonata 2.750 0.611 21 4.503 0.0040
 Water - Trichoderma 2.375 0.611 21 3.889 0.0159
 Water - Vacciplant 2.750 0.611 21 4.503 0.0040
 Bion - Helioterpene 0.250 0.611 21 0.409 0.9999
 Bion - LBG 0.000 0.611 21 0.000 1.0000
 Bion - Rhapsody 0.500 0.611 21 0.819 0.9900
 Bion - Sonata 0.125 0.611 21 0.205 1.0000
 Bion - Trichoderma -0.250 0.611 21 -0.409 0.9999
 Bion - Vacciplant 0.125 0.611 21 0.205 1.0000
 Helioterpene - LBG -0.250 0.611 21 -0.409 0.9999
 Helioterpene - Rhapsody 0.250 0.611 21 0.409 0.9999
 Helioterpene - Sonata -0.125 0.611 21 -0.205 1.0000
 Helioterpene - Trichoderma -0.500 0.611 21 -0.819 0.9900
 Helioterpene - Vacciplant -0.125 0.611 21 -0.205 1.0000
 LBG - Rhapsody 0.500 0.611 21 0.819 0.9900
 LBG - Sonata 0.125 0.611 21 0.205 1.0000
 LBG - Trichoderma -0.250 0.611 21 -0.409 0.9999
 LBG - Vacciplant 0.125 0.611 21 0.205 1.0000
 Rhapsody - Sonata -0.375 0.611 21 -0.614 0.9983
 Rhapsody - Trichoderma -0.750 0.611 21 -1.228 0.9142
 Rhapsody - Vacciplant -0.375 0.611 21 -0.614 0.9983
 Sonata - Trichoderma -0.375 0.611 21 -0.614 0.9983
 Sonata - Vacciplant 0.000 0.611 21 0.000 1.0000
 Trichoderma - Vacciplant 0.375 0.611 21 0.614 0.9983

Results are averaged over the levels of: time
Degrees-of-freedom method: kenward-roger
P value adjustment: tukey method for comparing a family of 8 estimates

## Pairwise comparisons to water (Dunnett)

summary(glht(res.lmer, linfct=mcp(product= "Dunnett", interaction_average= TRUE)))

Simultaneous Tests for General Linear Hypotheses

Multiple Comparisons of Means: Dunnett Contrasts


Fit: lmer(formula = Score ~ product + time + (1 | id) + (1 | repetition),
 data = genH1)

Linear Hypotheses:
 Estimate Std. Error z value Pr(>|z|)
Bion - Water == 0 -2.6250 0.6107 -4.298 < 1e-04 ***
Helioterpene - Water == 0 -2.8750 0.6107 -4.708 < 1e-04 ***
LBG - Water == 0 -2.6250 0.6107 -4.298 0.000129 ***
Rhapsody - Water == 0 -3.1250 0.6107 -5.117 < 1e-04 ***
Sonata - Water == 0 -2.7500 0.6107 -4.503 < 1e-04 ***
Trichoderma - Water == 0 -2.3750 0.6107 -3.889 0.000655 ***
Vacciplant - Water == 0 -2.7500 0.6107 -4.503 < 1e-04 ***
---
Signif. codes: 0 '***' 0.001 '**' 0.01 '*' 0.05 '.' 0.1 ' ' 1
(Adjusted p values reported -- single-step method)

# Trial 3: genotype K3

## Model:

res.lmerK3<- lmer(Score~product*time+(1|id)+(1|repetition), data=genK3)
Anova(res.lmerK3, test.statistic = "F")

Analysis of Deviance Table (Type II Wald F tests with Kenward-Roger df)

Response: Score
 F Df Df.res Pr(>F)
product 4.1186 7 21 0.005393 **
time 43.6046 1 24 7.881e-07 ***
product:time 2.1429 7 24 0.077587 .
---
Signif. codes: 0 '***' 0.001 '**' 0.01 '*' 0.05 '.' 0.1 ' ' 1

ranova(res.lmerK3)

ANOVA-like table for random-effects: Single term deletions

Model:
Score ~ product + time + (1 | id) + (1 | repetition) + product:time
 npar logLik AIC LRT Df Pr(>Chisq)
<none> 19 -57.306 152.61
(1 | id) 18 -60.488 156.97 6.3637 1 0.01165 *
(1 | repetition) 18 -57.708 151.42 0.8046 1 0.36973
---
Signif. codes: 0 '***' 0.001 '**' 0.01 '*' 0.05 '.' 0.1 ' ' 1

res.lmerK3<- lmer(Score~product+time+(1|id)+(1|repetition), data=genK3)
Anova(res.lmerK3, test.statistic = "F")

Analysis of Deviance Table (Type II Wald F tests with Kenward-Roger df)

Response: Score
 F Df Df.res Pr(>F)
product 4.1186 7 21 0.005393 **
time 34.6601 1 31 1.692e-06 ***
---
Signif. codes: 0 '***' 0.001 '**' 0.01 '*' 0.05 '.' 0.1 ' ' 1

ranova(res.lmerK3)

ANOVA-like table for random-effects: Single term deletions

Model:
Score ~ product + time + (1 | id) + (1 | repetition)
 npar logLik AIC LRT Df Pr(>Chisq)
<none> 12 -64.173 152.35
(1 | id) 11 -66.486 154.97 4.6249 1 0.03151 *
(1 | repetition) 11 -64.576 151.15 0.8046 1 0.36973
---
Signif. codes: 0 '***' 0.001 '**' 0.01 '*' 0.05 '.' 0.1 ' ' 1

### Postulates verification

shapiro.test(residuals(res.lmerK3))

Shapiro-Wilk normality test

data: residuals(res.lmerK3)
W = 0.98466, p-value = 0.6108


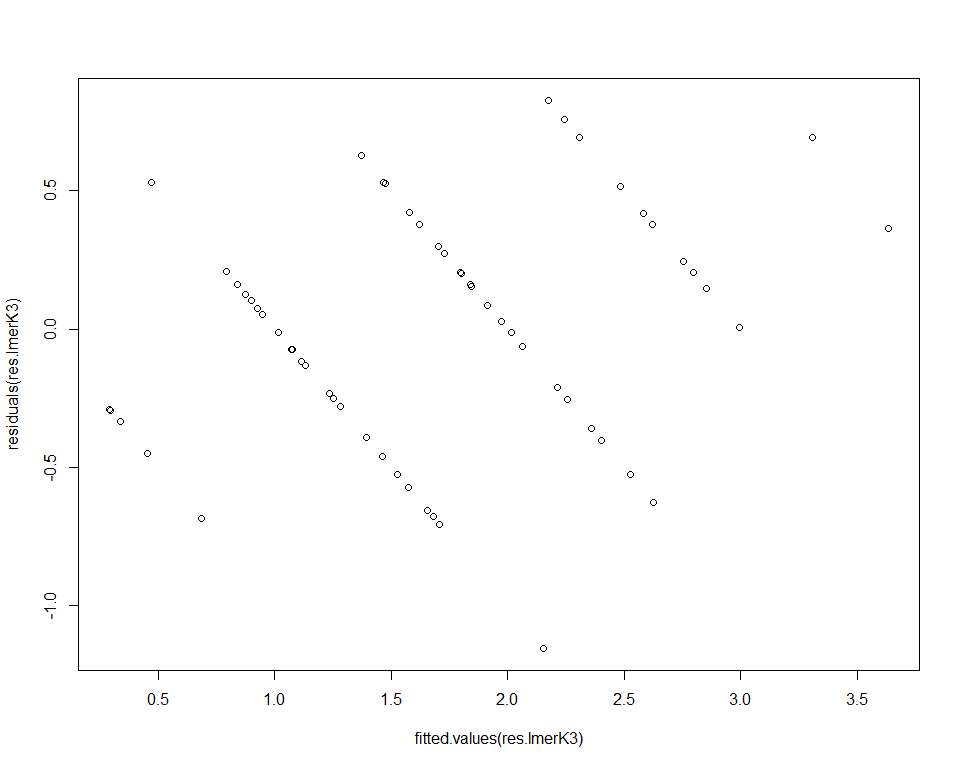


## Pairwise comparison (Tukey)

$`emmeans of product`
 product emmean SE df lower.CL upper.CL
 Bion 2.750 0.305 21.9 2.117 3.38
 Helioterpene 1.500 0.305 21.9 0.867 2.13
 LBG 1.750 0.305 21.9 1.117 2.38
 Rhapsody 1.625 0.305 21.9 0.992 2.26
 Sonata 0.875 0.305 21.9 0.242 1.51
 Trichoderma 1.875 0.305 21.9 1.242 2.51
 Vacciplant 1.125 0.305 21.9 0.492 1.76
 Water 2.125 0.305 21.9 1.492 2.76

Results are averaged over the levels of: time
Degrees-of-freedom method: kenward-roger
Confidence level used: 0.95

$`pairwise differences of product`
 1 estimate SE df t.ratio p.value
 Bion - Helioterpene 1.250 0.406 21 3.081 0.0868
 Bion - LBG 1.000 0.406 21 2.465 0.2629
 Bion - Rhapsody 1.125 0.406 21 2.773 0.1554
 Bion - Sonata 1.875 0.406 21 4.621 0.0031
 Bion - Trichoderma 0.875 0.406 21 2.157 0.4140
 Bion - Vacciplant 1.625 0.406 21 4.005 0.0123
 Bion - Water 0.625 0.406 21 1.540 0.7780
 Helioterpene - LBG -0.250 0.406 21 -0.616 0.9982
 Helioterpene - Rhapsody -0.125 0.406 21 -0.308 1.0000
 Helioterpene - Sonata 0.625 0.406 21 1.540 0.7780
 Helioterpene - Trichoderma -0.375 0.406 21 -0.924 0.9800
 Helioterpene - Vacciplant 0.375 0.406 21 0.924 0.9800
 Helioterpene - Water -0.625 0.406 21 -1.540 0.7780
 LBG - Rhapsody 0.125 0.406 21 0.308 1.0000
 LBG - Sonata 0.875 0.406 21 2.157 0.4140
 LBG - Trichoderma -0.125 0.406 21 -0.308 1.0000
 LBG - Vacciplant 0.625 0.406 21 1.540 0.7780
 LBG - Water -0.375 0.406 21 -0.924 0.9800
 Rhapsody - Sonata 0.750 0.406 21 1.848 0.5970
 Rhapsody - Trichoderma -0.250 0.406 21 -0.616 0.9982
 Rhapsody - Vacciplant 0.500 0.406 21 1.232 0.9128
 Rhapsody - Water -0.500 0.406 21 -1.232 0.9128
 Sonata - Trichoderma -1.000 0.406 21 -2.465 0.2629
 Sonata - Vacciplant -0.250 0.406 21 -0.616 0.9982
 Sonata - Water -1.250 0.406 21 -3.081 0.0868
 Trichoderma - Vacciplant 0.750 0.406 21 1.848 0.5970
 Trichoderma - Water -0.250 0.406 21 -0.616 0.9982
 Vacciplant - Water -1.000 0.406 21 -2.465 0.2629

Results are averaged over the levels of: time
Degrees-of-freedom method: kenward-roger
P value adjustment: tukey method for comparing a family of 8 estimates

## Pairwise comparison to water (Dunnett)

glht_res=summary(glht(res.lmerK3, linfct=mcp(product= "Dunnett", interaction_average= TRUE)))
glht_res

Simultaneous Tests for General Linear Hypotheses

Multiple Comparisons of Means: Dunnett Contrasts


Fit: lmer(formula = Score ~ product + time + (1 | id) + (1 | repetition),
 data = genK3)

Linear Hypotheses:
 Estimate Std. Error z value Pr(>|z|)
Bion - Water == 0 0.6250 0.4057 1.540 0.4753
Helioterpene - Water == 0 -0.6250 0.4057 -1.540 0.4756
LBG - Water == 0 -0.3750 0.4057 -0.924 0.8978
Rhapsody - Water == 0 -0.5000 0.4057 -1.232 0.7037
Sonata - Water == 0 -1.2500 0.4057 -3.081 0.0126 *
Trichoderma - Water == 0 -0.2500 0.4057 -0.616 0.9866
Vacciplant - Water == 0 -1.0000 0.4057 -2.465 0.0739 .
---
Signif. codes: 0 '***' 0.001 '**' 0.01 '*' 0.05 '.' 0.1 ' ' 1
(Adjusted p values reported -- single-step method)

# Trial 4: genotype H1

## Model:

res.lmer<- lmer(Score~product*time+(1|id)+(1|repetition), data=genH1)
Anova(res.lmer, test.statistic = "F")

Analysis of Deviance Table (Type II Wald F tests with Kenward-Roger df)

Response: Score
 F Df Df.res Pr(>F)
product 7.7882 7 21 0.0001106 ***
time 96.5711 1 24 6.903e-10 ***
product:time 1.0612 7 24 0.4173332
---
Signif. codes: 0 '***' 0.001 '**' 0.01 '*' 0.05 '.' 0.1 ' ' 1

ranova(res.lmer)

ANOVA-like table for random-effects: Single term deletions

Model:
Score ~ product + time + (1 | id) + (1 | repetition) + product:time
 npar logLik AIC LRT Df Pr(>Chisq)
<none> 19 -65.218 168.44
(1 | id) 18 -65.511 167.02 0.58508 1 0.44433
(1 | repetition) 18 -66.788 169.57 3.13822 1 0.07648 .
---
Signif. codes: 0 '***' 0.001 '**' 0.01 '*' 0.05 '.' 0.1 ' ' 1

res.lmer<- lmer(Score~product+time+(1|id)+(1|repetition), data=genH1)
Anova(res.lmer, test.statistic = "F")

Analysis of Deviance Table (Type II Wald F tests with Kenward-Roger df)

Response: Score
 F Df Df.res Pr(>F)
product 7.7882 7 21 0.0001106 ***
time 95.2546 1 31 5.709e-11 ***
---
Signif. codes: 0 '***' 0.001 '**' 0.01 '*' 0.05 '.' 0.1 ' ' 1

ranova(res.lmer)

ANOVA-like table for random-effects: Single term deletions

Model:
Score ~ product + time + (1 | id) + (1 | repetition)
 npar logLik AIC LRT Df Pr(>Chisq)
<none> 12 -71.084 166.17
(1 | id) 11 -71.388 164.78 0.60747 1 0.43574
(1 | repetition) 11 -72.653 167.31 3.13822 1 0.07648 .
---
Signif. codes: 0 '***' 0.001 '**' 0.01 '*' 0.05 '.' 0.1 ' ' 1

### Postulates verification

shapiro.test(residuals(res.lmer))

Shapiro-Wilk normality test

data: residuals(res.lmer)
W = 0.9886, p-value = 0.8222

plot(residuals(res.lmer)~ fitted.values(res.lmer))


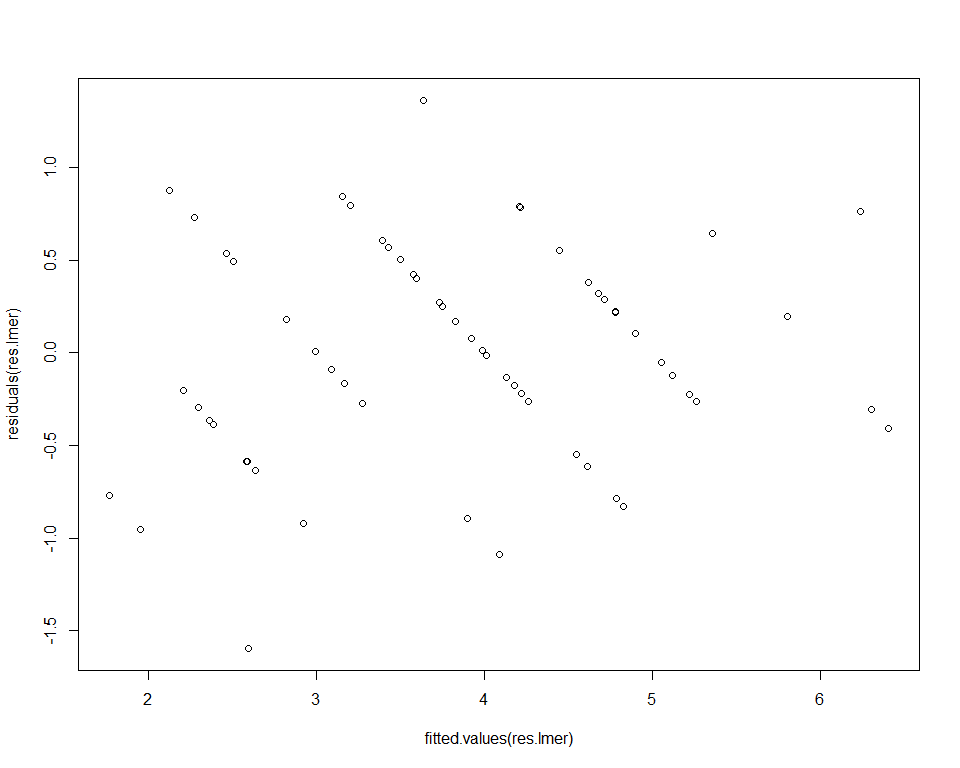


## Pairwise comparisons (Tukey)

emmeans(res.lmer, list(pairwise ~ product), adjust = "tukey")

$`emmeans of product`
 product emmean SE df lower.CL upper.CL
 Bion 3.00 0.319 16.4 2.33 3.67
 Helio 4.12 0.319 16.4 3.45 4.80
 LBG 4.00 0.319 16.4 3.33 4.67
 Rhapsody 3.75 0.319 16.4 3.08 4.42
 Sonata 3.25 0.319 16.4 2.58 3.92
 Trichoderma 3.12 0.319 16.4 2.45 3.80
 Vacciplant 4.12 0.319 16.4 3.45 4.80
 Water 5.38 0.319 16.4 4.70 6.05

Results are averaged over the levels of: time
Degrees-of-freedom method: kenward-roger
Confidence level used: 0.95

$`pairwise differences of product`
 1 estimate SE df t.ratio p.value
 Bion - Helio -1.125 0.389 21 -2.895 0.1242
 Bion - LBG -1.000 0.389 21 -2.573 0.2201
 Bion - Rhapsody -0.750 0.389 21 -1.930 0.5472
 Bion - Sonata -0.250 0.389 21 -0.643 0.9977
 Bion - Trichoderma -0.125 0.389 21 -0.322 1.0000
 Bion - Vacciplant -1.125 0.389 21 -2.895 0.1242
 Bion - Water -2.375 0.389 21 -6.111 0.0001
 Helio - LBG 0.125 0.389 21 0.322 1.0000
 Helio - Rhapsody 0.375 0.389 21 0.965 0.9747
 Helio - Sonata 0.875 0.389 21 2.251 0.3631
 Helio - Trichoderma 1.000 0.389 21 2.573 0.2201
 Helio - Vacciplant 0.000 0.389 21 0.000 1.0000
 Helio - Water -1.250 0.389 21 -3.216 0.0663
 LBG - Rhapsody 0.250 0.389 21 0.643 0.9977
 LBG - Sonata 0.750 0.389 21 1.930 0.5472
 LBG - Trichoderma 0.875 0.389 21 2.251 0.3631
 LBG - Vacciplant -0.125 0.389 21 -0.322 1.0000
 LBG - Water -1.375 0.389 21 -3.538 0.0340
 Rhapsody - Sonata 0.500 0.389 21 1.287 0.8939
 Rhapsody - Trichoderma 0.625 0.389 21 1.608 0.7407
 Rhapsody - Vacciplant -0.375 0.389 21 -0.965 0.9747
 Rhapsody - Water -1.625 0.389 21 -4.181 0.0083
 Sonata - Trichoderma 0.125 0.389 21 0.322 1.0000
 Sonata - Vacciplant -0.875 0.389 21 -2.251 0.3631
 Sonata - Water -2.125 0.389 21 -5.468 0.0004
 Trichoderma - Vacciplant -1.000 0.389 21 -2.573 0.2201
 Trichoderma - Water -2.250 0.389 21 -5.789 0.0002
 Vacciplant - Water -1.250 0.389 21 -3.216 0.0663

Results are averaged over the levels of: time
Degrees-of-freedom method: kenward-roger
P value adjustment: tukey method for comparing a family of 8 estimates

## Pairwise comparisons to water (Dunnett)

summary(glht(res.lmer, linfct=mcp(product= "Dunnett", interaction_average= TRUE)))

Simultaneous Tests for General Linear Hypotheses

Multiple Comparisons of Means: Dunnett Contrasts


Fit: lmer(formula = Score ~ product + time + (1 | id) + (1 | repetition),
 data = genH1)

Linear Hypotheses:
 Estimate Std. Error z value Pr(>|z|)
Bion - Water == 0 -2.3750 0.3886 -6.111 < 0.001 ***
Helio - Water == 0 -1.2500 0.3886 -3.216 0.00801 **
LBG - Water == 0 -1.3750 0.3886 -3.538 0.00260 **
Rhapsody - Water == 0 -1.6250 0.3886 -4.181 < 0.001 ***
Sonata - Water == 0 -2.1250 0.3886 -5.468 < 0.001 ***
Trichoderma - Water == 0 -2.2500 0.3886 -5.789 < 0.001 ***
Vacciplant - Water == 0 -1.2500 0.3886 -3.216 0.00832 **
---
Signif. codes: 0 '***' 0.001 '**' 0.01 '*' 0.05 '.' 0.1 ' ' 1
(Adjusted p values reported -- single-step method)

# Trial 4: genotype K3

## Model

res.lmerK3<- lmer(Score~product*time+(1|id)+(1|repetition), data=genK3)
Anova(res.lmerK3, test.statistic = "F")

Analysis of Deviance Table (Type II Wald F tests with Kenward-Roger df)

Response: Score
 F Df Df.res Pr(>F)
product 7.2469 7 21 0.0001815 ***
time 336.1036 1 24 1.278e-15 ***
product:time 4.8325 7 24 0.0016491 **
---
Signif. codes: 0 '***' 0.001 '**' 0.01 '*' 0.05 '.' 0.1 ' ' 1

ranova(res.lmerK3)

ANOVA-like table for random-effects: Single term deletions

Model:
Score ~ product + time + (1 | id) + (1 | repetition) + product:time
 npar logLik AIC LRT Df Pr(>Chisq)
<none> 19 -41.943 121.89
(1 | id) 18 -43.198 122.39 2.51000 1 0.1131
(1 | repetition) 18 -41.944 119.89 0.00207 1 0.9637

### Postulates verification

shapiro.test(residuals(res.lmerK3))

Shapiro-Wilk normality test

data: residuals(res.lmerK3)
W = 0.96458, p-value = 0.06309


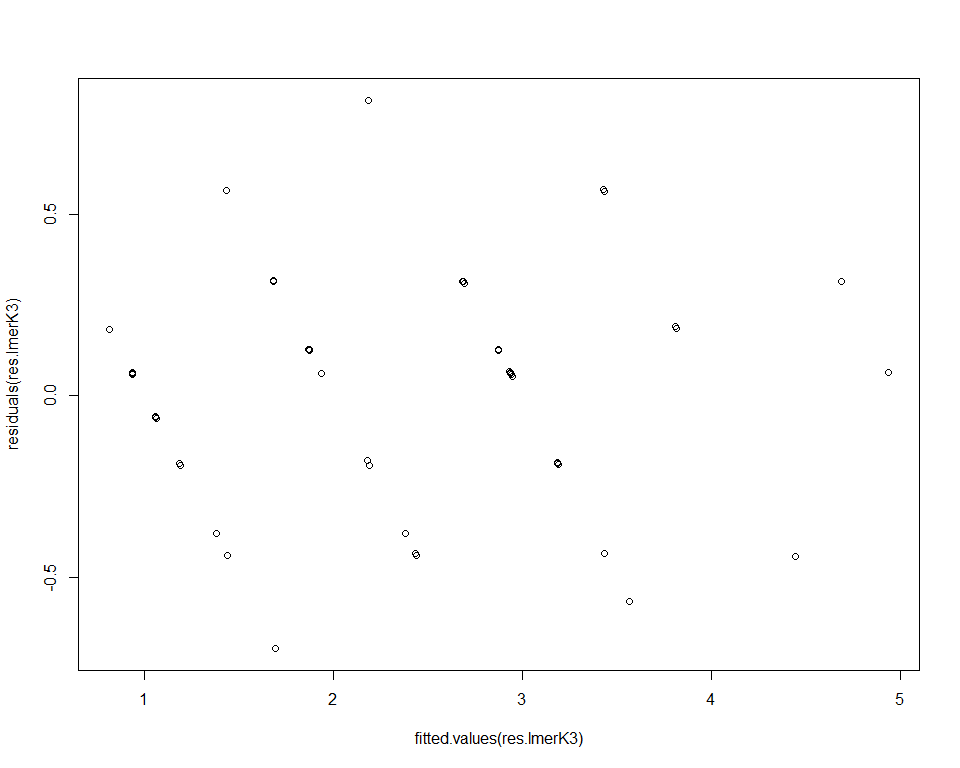


## Pairwise comparisons (Tukey)

summary(glht(res.lmerK3, linfct=mcp(product= "Tukey", interaction_average= TRUE)))

Simultaneous Tests for General Linear Hypotheses

Multiple Comparisons of Means: Tukey Contrasts


Fit: lmer(formula = Score ~ product * time + (1 | id) + (1 | repetition),
 data = genK3)

Linear Hypotheses:
 Estimate Std. Error z value Pr(>|z|)
Helio - Bion == 0 1.250e-01 2.717e-01 0.46 0.99981
LBG - Bion == 0 -3.442e-15 2.717e-01 0.00 1.00000
Rhapsody - Bion == 0 -3.553e-15 2.717e-01 0.00 1.00000
Sonata - Bion == 0 2.500e-01 2.717e-01 0.92 0.98426
Trichoderma - Bion == 0 -3.636e-15 2.717e-01 0.00 1.00000
Vacciplant - Bion == 0 5.000e-01 2.717e-01 1.84 0.59246
Water - Bion == 0 1.500e+00 2.717e-01 5.52 < 0.001 ***
LBG - Helio == 0 -1.250e-01 2.717e-01 -0.46 0.99981
Rhapsody - Helio == 0 -1.250e-01 2.717e-01 -0.46 0.99981
Sonata - Helio == 0 1.250e-01 2.717e-01 0.46 0.99981
Trichoderma - Helio == 0 -1.250e-01 2.717e-01 -0.46 0.99981
Vacciplant - Helio == 0 3.750e-01 2.717e-01 1.38 0.86661
Water - Helio == 0 1.375e+00 2.717e-01 5.06 < 0.001 ***
Rhapsody - LBG == 0 -1.110e-16 2.717e-01 0.00 1.00000
Sonata - LBG == 0 2.500e-01 2.717e-01 0.92 0.98425
Trichoderma - LBG == 0 -2.220e-16 2.717e-01 0.00 1.00000
Vacciplant - LBG == 0 5.000e-01 2.717e-01 1.84 0.59260
Water - LBG == 0 1.500e+00 2.717e-01 5.52 < 0.001 ***
Sonata - Rhapsody == 0 2.500e-01 2.717e-01 0.92 0.98424
Trichoderma - Rhapsody == 0 -1.110e-16 2.717e-01 0.00 1.00000
Vacciplant - Rhapsody == 0 5.000e-01 2.717e-01 1.84 0.59267
Water - Rhapsody == 0 1.500e+00 2.717e-01 5.52 < 0.001 ***
Trichoderma - Sonata == 0 -2.500e-01 2.717e-01 -0.92 0.98426
Vacciplant - Sonata == 0 2.500e-01 2.717e-01 0.92 0.98425
Water - Sonata == 0 1.250e+00 2.717e-01 4.60 < 0.001 ***
Vacciplant - Trichoderma == 0 5.000e-01 2.717e-01 1.84 0.59252
Water - Trichoderma == 0 1.500e+00 2.717e-01 5.52 < 0.001 ***
Water - Vacciplant == 0 1.000e+00 2.717e-01 3.68 0.00573 **
---
Signif. codes: 0 '***' 0.001 '**' 0.01 '*' 0.05 '.' 0.1 ' ' 1
(Adjusted p values reported -- single-step method)

## Pairwise comparisons (Dunnett)

summary(glht(res.lmerK3, linfct=mcp(product= "Dunnett", interaction_average= TRUE)))

Simultaneous Tests for General Linear Hypotheses

Multiple Comparisons of Means: Dunnett Contrasts


Fit: lmer(formula = Score ~ product * time + (1 | id) + (1 | repetition),
 data = genK3)

Linear Hypotheses:
 Estimate Std. Error z value Pr(>|z|)
Bion - Water == 0 -1.5000 0.2717 -5.52 < 1e-04 ***
Helio - Water == 0 -1.3750 0.2717 -5.06 < 1e-04 ***
LBG - Water == 0 -1.5000 0.2717 -5.52 < 1e-04 ***
Rhapsody - Water == 0 -1.5000 0.2717 -5.52 < 1e-04 ***
Sonata - Water == 0 -1.2500 0.2717 -4.60 < 1e-04 ***
Trichoderma - Water == 0 -1.5000 0.2717 -5.52 < 1e-04 ***
Vacciplant - Water == 0 -1.0000 0.2717 -3.68 0.00149 **
---
Signif. codes: 0 '***' 0.001 '**' 0.01 '*' 0.05 '.' 0.1 ' ' 1
(Adjusted p values reported -- single-step method)
